# Strategies to Facilitate Improved Recruitment, Development, and Retention of the Rural and Remote Medical Workforce: A Scoping Review

**DOI:** 10.34172/ijhpm.2021.160

**Published:** 2021-11-15

**Authors:** Farah Noya, Sandra Carr, Kirsty Freeman, Sandra Thompson, Rhonda Clifford, Denese Playford

**Affiliations:** ^1^Division of Health Professions Education, School of Allied Health, University of Western Australia, Perth, WA, Australia.; ^2^Duke National University Singapore Medical School, Singapore, Singapore.; ^3^Western Australian Centre for Rural Health, The University of Western Australia, Perth, WA, Australia.; ^4^School of Allied Health, University of Western Australia, Perth, WA, Australia.; ^5^The Rural Clinical School of WA, School of Medicine, The University of Western Australia, Perth, WA, Australia.

**Keywords:** Recruitment and Retention Strategies, Rural and Remote, Medical Workforce Shortage, High-Income Countries, Low- and Middle-Income Countries

## Abstract

**Background:** Medical workforce shortages in rural and remote areas are a global issue. High-income countries (HICs) and low- and middle-income countries (LMICs) seek to implement strategies to address this problem, regardless of local challenges and contexts. This study distilled strategies with positive outcomes and success from international peer-reviewed literature regarding recruitment, retention, and rural and remote medical workforce development in HICs and LMICs.

**Methods:** The Arksey and O’Malley scoping review framework was utilised. Articles were retrieved from electronic databases Medline, Embase, Global Health, CINAHL Plus, and PubMed from 2010-2020. The Preferred Reporting Items for Systematic Review and Meta-Analysis Protocols (PRISMA-P) guideline was used to ensure rigour in reporting the methodology in the interim, and PRISMA extension for scoping review (PRISMA-ScR) was used as a guide to report the findings. The success of strategies was examined against the following outcomes: for recruitment - rural and remote practice location; for development - personal and professional development; and for retention - continuity in rural and remote practice and low turnover rates.

**Results:** Sixty-one studies were included according to the restriction criteria. Most studies (n=53; 87%) were undertaken in HICs, with only eight studies from LMICs. This scoping review found implementation strategies classified as Educational, Financial, and Multidimensional were successful for recruitment, retention, and development of the rural and remote medical workforce.

**Conclusion:** This scoping review shows that effective strategies to recruit and retain rural and remote medical workforce are feasible worldwide despite differences in socio-economic factors. While adjustment and adaptation to match the strategies to the local context are required, the country’s commitment to act to improve the rural medical workforce shortage is most critical.

## Background

 Shortages of medical personnel and maldistribution of the workforce remain critical problems for many rural and remote communities and contribute to disparities in the health between rural and urban populations. Many factors contribute to the low number of medical personnel in rural and remote areas. In addition to the rural and remote nature of areas being a disincentive for medical personnel choosing to live and work there,^1-4^ when compared with urban areas, rural areas have less infrastructure, facilities and amenities, difficulties with internet and poorer access to education.^1-4^ Additionally, the opportunity costs of rural practice include lost income because of limited opportunities for private practice in rural areas and additional housing costs that may occur with maintaining a residence in an urban area for children’s education and a spouse’s job.^5,6^ Some studies in several low- and middle-income countries (LMICs) report that doctors prefer employment in a rural area only in specific circumstances that reflect their interests.^7-10^

 Aiming to provide evidence-based global recommendations to address the problem of rural workforce shortage, in 2010, the World Health Organization (WHO) recommended a global policy on “Increasing access to health workers in remote and rural areas through improved retention.”^5^ The recommendation included educational approaches, regulation, financial inducements, and personal and professional support.^5^

 Research has shown that medical school social accountability plays an essential role in facilitating a sustainable rural and remote workforce within the education domain.^11-22^ As defined by WHO, medical school social accountability is “the obligation to direct their education, research, and service activities toward addressing the priority health concerns of the community, the region, or nation they have a mandate to serve.”^23^ Social accountability approaches implemented by medical schools include, but are not limited to, recruiting students of rural origin^12,14,20^ and training including relevant rural learning experiences.^15,21,22^ These actions have been proved to increase medical graduates’ intentions to work in rural and remote areas and the recruitment of rural workforce.^12,14,20^ Another WHO recommendation for improved rural workforce retention is through regulatory interventions.^5^ Government interventions include creating conducive conditions for health providers, rapid training to fulfil the workforce demand, optimizing the use of bonded service, and allocating educational grants to compulsory rural assignments.^5^ Financial inducement is another factor influencing recruitment and retention of the medical workforce, especially in LMICs.^5,6^ This stimulus includes financial bonuses, in-kind benefits (subsidised or free housing or vehicle), and other benefits that make working in rural areas more attractive and offset other costs and losses of working rurally.

 Since the publication of the WHO global recommendations on the recruitment and retention of the health workforce, more LMICs have increased their focus on approaching this problem. A number of Medical Schools and Government agencies in LMICs have implemented strategies aimed at improving recruitment and retention.^24-26^ A preliminary search of MEDLINE, the Cochrane Database of Systematic Reviews and the *JBI Database of Systematic Reviews and Implementation Reports *identified no existing systematic reviews on the topic of interest or protocols flagging a review on the medical workforce in rural areas and remote areas internationally was underway. While there are existing reviews on rural workforce recruitment and retention, most either do not focus exclusively on the medical workforce or focus on high-income countries (HICs) such as the United States, Canada and Australia which have very different medical education and healthcare systems from LMICs.^27-30^ Scoping reviews map the literature on a particular topic and provide an opportunity to identify key concepts and gaps, clarifying conceptual boundaries and definitions relating to a particular topic. Therefore, to ensure a comprehensive scoping review, it is important to include and compare the experiences of both HICs and LMICs in this review, irrespective of their medical education and healthcare system.

 In a recent review focusing on Asia Pacific LMICs,^31^ the outcomes included intention and preference to practising rurally in the future. We aimed only to include studies with the actual (current) location of practice as the outcome. This review synthesizes evidence from peer-reviewed and grey literature describing recruitment, development, and retention of the rural medical workforce in both HICs and LMICs. It includes strategies empirically associated with success, defined as the improvement of medical workforce in practice in rural and remote areas. The review aims to assist medical schools and policy-makers internationally in adopting strategies to improve recruitment and retention of their local rural and remote medical workforce and will be particularly useful for countries where health programs are limited by medical workforce shortages in rural and remote areas. The significance of this review relates to its capacity to describe evidence-informed approaches that have potential success in addressing the global issues surrounding the rural and remote medical workforce. To meet the objectives, we ask the following questions regarding the rural and remote medical workforce:

1. What factors have been shown associated with improved recruitment, development, and retention?
2. What strategies/approaches have been implemented to improve recruitment, development, and retention?
3. What is the evidence of the success of these approaches?
4. What are the similarities and differences between approaches implemented in HICs and LMICs?

## Methods

###  Protocol and Registration

 The protocol of this study has been registered with the Open Science Framework (https://osf.io/e83hp/) and published in the *International Journal of Health Policy and Management* Volume 10, Issue 1, January 2021, Pages 22-28.^32^

 This scoping review was conducted in accordance with the Arksey and O‘Malley framework for scoping reviews.^33^ This review follows the relevant aspects of the Preferred Reporting Items for Systematic Review and Meta-Analysis Protocols (PRISMA-P) guidelines^34^ to ensure rigour in reporting the methodology in the interim. The PRISMA extension for scoping review (PRISMA-ScR)^35^ used as a guide to ensure the robustness in reporting the findings of scoping reviews (Supplementary file 1).

###  Eligibility Criteria 

 The research question was developed as a broad framing of the population (ie, medical workforce), the concept (ie, recruitment, development, and retention of the workforce) and the context (ie, rural and remote areas in HICs and LMICs, regardless the definition of rurality and rural background used in one country) to be explored and mapped to the objectives of the review.

###  Information Sources

 Medline, Embase, Global Health, CINAHL Plus, and PubMed, which comprehensively capture relevant health literature were searched. The initial search query was developed for Medline (Ovid, including in-process and other non-indexed citation) with the advantage of using the MeSH terms to index the citations and a shared platform with Embase for a quicker translation of search strategy. Sources of unpublished studies and grey literature were searched using the Google Scholar - Advanced Search tool.

###  Search Strategy

 The search strategy sourced both published and unpublished studies. An initial limited search of MEDLINE and CINAHL Plus was undertaken to identify relevant articles. The words in the titles and abstracts of relevant articles and their index terms were used to develop a full search strategy for each relevant database (Box 1, Supplementary file 2). This search strategy, was adapted for each included bibliographic or information source, including keywords and index terms *Rural Population/*Rural Health/rural ares*, rural communit*, rural practice*, remote area*, remote communit*, remote practice* AND medical workforce, medical graduate*, medical worker*, medical profession* AND recruitment strateg*, “recruit and retain,” “recruitment and retention,” retention strateg*. The reference list of all studies selected for critical appraisal was then scanned for additional studies. The search strategy relates to the Participants, Concept, and Context of the medical workforce; recruitment, development, and retention strategy; and rural and remote areas in both HICs and LMICs. A total of 3283 articles were identified for inclusion.

**Box 1. Search Terms**

** Rural And Remote Areas**

*Rural Population/*Rural Health Services/*Rural Health/rural area*, rural communit*, rural location*, rural practice*, remote area*, remote communit*, remote location*, remote practice*, underserved area*, underserved location*, underserved communit*, geographically isolated area*, geographically isolated communit*, island* communit*, small island* communit*, remote island* communit*, poorly served area*, poorly served communit*, underserviced area*, “rural and remote area*,” *Medically Underserved Area/

** Medical Workforce**

*general practitioners/or *physicians, family/or *physicians, primary care/*general practice/or *family practice/exp Medical Staff/medical doctor.mp, Medical officer*, medical worker*, medical profession*, medical workforce, medical graduate*, health centre*, medical centre*, international medical graduate*, foreign medical graduate*, communit* medicine, *Workforce/*Physicians/sn, sd

** Recruitment, Development And Retention Strategies**

*personnel selection/or *“personnel staffing and scheduling”/or *personnel turnover/or *staff development/or *strikes, employee/or *work engagement/or *workplace/*Job Satisfaction/*Personnel Loyalty/*Personal Satisfaction/*Career Choice/*Career Mobility/personnel recruitment, sustainable rural practice, (sustain* adj3 employ*), (attract* adj3 employ*), personnel shortage*, workforce shortage*, “attract and retain,” “recruit and retain,” “recruitment and retention,” “recruiting and retaining,” (workforce adj3 maldistribut*), under distribut*, (commit* adj3 employ*), *Motivation/ph, sn [Physiology, Statistics & Numerical Data], (intrinsic adj3 motivat*), (hire* adj3 staff), improv* access, engag* employ*, interest* employ*, (attract* adj3 employ*), encourage* employ*, work* satisfaction*, (career adj3 advance*), Unmet Need*, workforce need*, recruitment strateg*, (retention adj2 strateg*), career development, (plan* adj5 workforce), *health plan implementation/or *health priorities/*Health Policy/government initiative*, support structure, practical model*, (rural adj3 package*), alternative model*, locum service*, locum support, (compulsory adj3 assignment*), compulsory service*, bond* scheme*, bond* service*, vacancy rate*, utilization of service*, duration of service*, *Program Evaluation/*Survival Analysis/*regression analysis/factor* impact*, polic* analysis, polic* initiativ*.ti,ab, *physician incentive plans/or *“salaries and fringe benefits”/*Remuneration/financial incentive*, financial inducement*, monetary incentive*, (non-financial adj3 inducement*), non-monetary incentive*, incentiv* measure*, incentiv* polic*, *Socioeconomic factors/exp Education, Medical/faculty development, professional development, rural exposure, rural learning experiences, rural scholarship, *Training Support/educational grant*, *Schools, Medical/community participation, social accountabilit*, *Social Responsibility/*Community-Institutional Relations/

###  Study Selection

 Following the search, all identified citations were collated and exported into EndNote format or using the Research Information Systems text format. These citations were then transferred to a systematic review management software Covidence.^36^ A total of 1391 titles and abstracts were screened for assessment of their relevance, each by two reviewers (FN, KF). To be relevant for full-text review, the title and abstract needed to focus on the medical workforce, recruitment, retention, and/or development of the workforce in a rural and remote setting, and describe the approach or strategy that was effectively used implemented.

 This scoping review included randomized controlled trials, non-randomized controlled trials, before and after studies, and interrupted time-series studies. In addition, analytical observational studies, including prospective and retrospective cohort studies, case-control studies, and analytical cross-sectional studies, were also included. This review also considered descriptive observational study designs, including case series, case studies, and descriptive cross-sectional studies for inclusion. Qualitative studies, including action research, were also considered. Studies published from January 1, 2010 to November 10, 2020 were included to identify up to date evidence from the last decade.

 Each of 173 articles identified from the title and abstract screening was selected for full-text review and assessed independently by two reviewers (FN, KF) against the inclusion criteria and for their focus on the medical workforce, recruitment, retention, workforce development, and description of the implemented approach or strategy. Thus, inclusion criteria to guide the assessment of each article required articles that:

1. Focused on the medical workforce in rural and/or remote settings.
2. Described rural and/or remote areas as the actual workplace of the medical workforce, not only perceptions/intentions/interests/career choices without evidence of participant doctors’ rural practice.
3. Described the recruitment or development, or retention of the workforce in the rural and remote areas.
4. Described/discussed the approach/strategy to improve recruitment or development or retention of the rural medical workforce.

Articles had to meet all four criteria to be included in the full-text review. Based on these criteria, articles were excluded before data extraction when they had:

1. No clear evidence of outcomes,
2. No positive outcomes (excluded as we were searching for effective strategies only),
3. No discussion of strategies/approaches that were effective for recruiting, developing or retaining a medical workforce in a rural and remote setting, or
4. The full text was in a language other than English.

###  Data Collection Process

 Data were extracted from the 61 papers meeting the inclusion criteria by FN, then all reviewed and agreed by KF, collated into an Excel spreadsheet. This data extracted included specific details about the population, concept, context, study methods, and key findings relevant to the review objective. A charting table is provided (Supplementary file 3).

###  Data Items

 Data items collected in this study were targeted surrounding Population, Concept, Context, and Expected Outcomes as follow:

*Population*: rural and remote medical workforce

*Concept*: recruitment, development, retention of rural and remote workforce

*Context*: HICs and LMICs

*Expected outcomes*: included but was not limited to these reported outcomes: for recruitment – rural and remote practice location; for development – personal and professional development; and for retention – continuity in rural and remote practice and low turnover rates.

####  Summary Measures 

 Comparison of results were made irrespective of the definitions of rurality in a study. Although there is Degree of Urbanization, a United Nation recommendation on the method to delineate cities, urban and rural areas for international statistical comparisons,^37^ it has not been used globally, thus the definition of rurality and rural background is different across studies, made it problematic for comparison. For the purpose of the study, we looked out the success of a strategy by their likelihood/odds ratios (ORs) to the outcomes, regardless of the definition for rurality and rural background.

###  Synthesis of Results

 A descriptive qualitative analysis was carried out for the included studies, and the findings from the included studies were analysed thematically.^38,39^ Our framework for analysis can be seen in Table 1. First, we assigned the themes from the findings to the three main concepts guiding the study: recruitment, development, and retention. Under these concepts, strategies and sub-strategies derived from the studies were listed. The strategies derived from the articles were aligned with the three categories documented in the WHO Global Policy Recommendation. That is, we categorised the strategies as: Education, Policy, and Financial Incentives. Multidimensional was added as the fourth category to capture instances where strategies were bundled or combined. The strategies and sub-strategies were then aligned to three levels at which they were implemented: university level, government or non-government levels and multilevel (There was a collaboration between university and government/non-government organisation).

**Table 1:** The Framework of Analysis and Summary of the Effective Strategies for Rural Medical Workforce Recruitment, Development and Retention

		**Concept**					
		**Recruitment**		**Development**		**Retention**	
**Level of Strategy**	**Type of Strategy**	**n = 50**	**Author, Year, Country**	**n = 9**	**Author, Year, Country**	**n = 16**	**Author, Year, Country**
University Medical School level	**Educational**	**37**		**1**		**4**	
	*Student selection*	6	Playford, 2019, Australia^40^; Hogenbirk, 2015, Canada^41^; Ray, 2015, Australia^42^; Puddey, 2015, Australia^43^; Rabinowitz, 2012b, USA^44^; Rabinowitz, 2012a, USA^45^				
	*Rural exposure/rural immersion*	13	Campbell, 2019, Australia^46^; McGirr, 2019, Australia^47^; Moore, 2018, Australia^48^; O'Sullivan, 2018, Australia^49^; Kwan, 2017, Australia^50^; Petrany, 2017, USA^51^; Playford, 2017, Australia^14^; Crump, 2016, USA^52^; Myhre, 2016, Canada^53^; Runge, 2016, Australia^54^; Playford, 2015, Australia^55^; Shires, 2015, Australia^56^; Playford, 2014, Australia^15^			1	Boonluksiri, 2018, Thailand^57^
	*Rural training *	1	Jamieson, 2014, Canada^58^			1	Morken, 2018, USA^59^
	*Comprehensive medical school program*	17	McGrail, 2018, Australia^60^; Rourke, 2018, Canada^61^; Woolley, 2018, Phillipines^62^; Fuglestad, 2017, USA^63^; Halili, 2017, Phillipines^64^; Mian, 2017, Canada^65^; Playford, 2017, Australia^66^; Siega-Sur, 2017, Phillipines^67^; Wenghofer, 2017, Canada^68^; Wendling, 2016, USA^69^; Woolley, 2016, Australia^70^; Woolley, 2014, Australia^71^; MacDowell, 2013, USA^72^; Quinn, 2011, USA^73^; Rabinowitz, 2011, USA^74^; Glasser, 2010, USA^75^; Woolley, 2017, Australia^76^			2	MacDowell, 2013, USA^72^; Glasser, 2010, USA^75^
	*Professional development*			1	Vyas, 2014, India^77^		
Government/non-government organisation level	**Educational **	**2**		**3**		**4**	
	*Rural training *	2	Orda, 2017, Australia^78^; McGrail, 2016, Australia^79^	1	Orda, 2017, Australia^78^	3	Orda, 2017, Australia^78^; Robinson, 2013, Australia^80^; Straume, 2010b, Norway^81^
	*Professional development*			2	Gorsche, 2012, Canada^82^; Martin, 2019, Australia^83^	1	Gorsche, 2012, Canada^82^
	**Policy**					**1**	Mowat, 2017, Canada^85^
	**Financial incentives**	**2**				**1**	
	*Obligatory time commitment*	1	Opoku, 2015, USA^86^			1	Opoku, 2015, USA^86^
	*Bonded scholarships*	1	Lewis, 2016, Australia^87^				
	**Multidimensional**	**4**	Chevillard, 2019, France^88^; Pereira, 2016, Brazil^84^; Kehlet, 2015, Norway^89^; Pena, 2010, Chile^90^	**3**	Straume, 2010, Norway^91^; Pena, 2010, Chile^90^; Kehlet, 2015, Norway^89^	**3**	Straume, 2010a, Norway^91^; Pena, 2010, Chile^90^; Kehlet, 2015, Norway^89^
Multilevel/Collaboration (university medical school partnership with government/ foundation)	**Educational**	**2**		**1**		**1**	
	*Student selection*	1	Beauchamp, 2013, Canada^92^				
	*Rural exposure/rural immersion*			1	Bing-You, 2014, USA^93^		
	*Comprehensive medical school program*	1	Matthews, 2015, New Zealand^94^			1	Pagaiya, 2015, Thailand^95^
	**Financial incentives**	**1**					
	*Obligatory time commitment*	1	Matsumoto, 2010, Japan^96^				
	**Multidimensional**	**2**	MacVicar, 2016, Scotland^97^; Reid, 2019, USA^98^	**1**	MacVicar, 2016, Scotland^97^	**2**	Arora, 2017, Thailand^99^; MacVicar, 2016, Scotland^97^

 Tables were used for descriptive numerical analysis and distribution of the studies included in the review, the research methods used, and the geographical distribution of the studies. Tables also used for categorisation of strategies improved recruitment, development and retention of the rural and remote medical workforce,

 The PRISMA^34^ diagram was utilized along with a final report of the review as per the PRISMA-ScR guidelines.^35^

## Results

###  Study Selection

 We identified 3283 articles using specific search terms in CINAHL, EMBASE, Global Health, Medline and PubMed. After excluding duplicates, articles not meeting the inclusion criteria and articles without adequate information, 61 studies were included (Figure 1).

**Figure:**
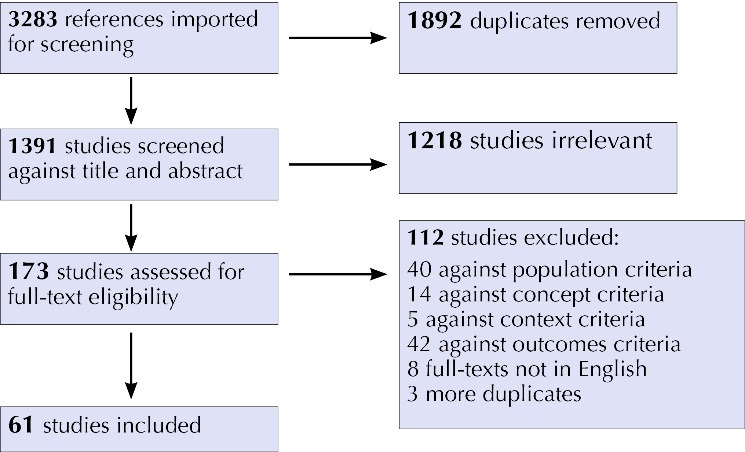
PRISMA Flow Chart of Included Studies. Abbreviation: PRISMA, Preferred Reporting Items for Systematic Review and Meta-Analysis.

###  Study Characteristics

 As can be seen in Table 2, there were 44 studies (72%) published within the last five years, particularly in 2017 (n = 13; 21%). Australia (n = 24; 39%), the USA (n = 13; 21%) and Canada (n = 8; 13%) were the three countries predominating in reported studies. While 10 (14%) of the studies were conducted in Asian countries (Japan, The Philippines, Thailand and India), only 5 (8%) were conducted in European countries (France, Norway, and Scotland) and 23 (38%) were conducted in American countries (The United States, Canada, Chile, and Brazil). Twenty-five (41%) of the studies were conducted in Australia and New Zealand. Most of the studies (n = 53; 87%) were conducted in HICs, with only eight (13%) from LMICs.

**Table 2:** Distribution of Studies Examining Rural and Remote Medical Workforce by Year and Country

**Published **	**n**	**%**
Year		
2010	5	8
2011	2	3
2012	2	3
2013	3	5
2014	5	8
2015	9	15
2016	9	15
2017	13	21
2018	7	11
2019	6	10
2020	0	0
**Total**	**61**	**100**
Country		
Australia	24	39
Brazil	1	2
Canada	8	13
Chile	1	2
France	1	2
India	1	2
Japan	1	2
New Zealand	1	2
Norway	3	5
Philippines	3	5
Scotland	1	2
Thailand	3	5
USA	13	21
**Total**	**61**	**100**

###  Methodological Aspects of the Studies

 Almost all the papers were research articles (n = 49; 80%) with the remainder being project or policy reports (n = 10; 16%) and program evaluations (n = 2; 3%). Most of the 61 studies used quantitative data analysis (n = 50; 82%), and the remainder applied mixed methods (n = 8; 13%) or qualitative analysis procedures (n = 3; 5%). Among the quantitative studies, 20 (33%) were longitudinal studies collecting data from a series of cohorts, whilst 22 (36%) used cross-sectional surveys and database tracking. The qualitative and mixed methods studies included approaches such as in-depth and semi-structured interviews, focus group discussions, surveys, case studies and observational questionnaires applying thematic and content analysis procedures.

###  Concept and Outcomes of the Studies

 Most of the studies (n = 43, 70%) only focused on recruitment. Seven (12%) focussed on retention alone, and only two focused on the development of the rural workforce. Three studies (5%) discussed recruitment and retention together, two (3%) discussed retention and development and four (7%) discussed development, together with recruitment and retention of the rural and remote medical workforce.

 All studies focusing on recruitment reported the current practice location being in rural and remote areas as their outcome. Regarding retention, continuity of practice in rural and remote medicine was used as the main outcome (n = 15), followed by low turnover (n = 1). The development concept included skills and professional development as the main outcome of interest (Supplementary file 4).

###  Definition of Rurality and Rural Background

 Fifty-six (88%) studies delineated their measure for how rurality was defined, and the remainder did not. Of those reporting an explicit definition of the term: “rural,” most (n = 33, 59%) used their country’s local definition/s. One quarter (n = 14) created their definitions based on the literature, and the rest simply referred to given geography as “rural” without any empirical reference. Although the definition of rural varies between countries or within a country, the use of population size as 10 000 per area was commonly used in the United States and Canada and more recently in Australia using the Modified Monash Model criteria(Supplementary file 5).^14,15,40-44,46-55,57-60,62,63,65,67-70,72-79,82-84,87-92,95,97,99-102^

 Rural background, rural exposure and rural immersion were the most dominant themes derived from the studies. Slightly different definitions across studies were identified, although the general meaning remained the same. Rural background was generally defined as having spent considerable time during a person’s formative years living in a rural setting included originating from targeted rural and remote areas, whether using a national classification system or not. Some studies used duration of rural living prior to commencing medical school (again, without an empirical value), and others added parents’ rural origin.^14,43,99^ Some studies defined rural background as “rural upbringing” and described it simply as ‘growing up in a rural area.’^45^ In the Philippines, preferential selection was based on a local definition of a rural community, also being from lower socio-economic strata.^64,67^

 Rural exposure and rural immersion have a common definition as experienced rural, but slightly different in the period of time being. Rural exposure means opportunities to visit and see rural communities and health settings; it can be for relatively short periods. Rural immersion means living and working in a rural environment, where a person is considered part of the community for a period of time. However, these two terms were used interchangeably in many studies. We tend to use rural immersion as most strategies relating to these themes described medical students and doctors living and working rurally during their clerkship, internship and training. For distributed or decentralised postgraduate training, we use the term “rural training.”

###  Recruitment

 As can be seen in Table 3, there were single and multiple factors reported as increasing the likelihood of recruitment to rural practice. Whether bivariate or logistic regression analysis was used, rural background^15,41,50,52,60,63,96^ and rural immersion^15,50,52,58,60,96^ were the two single factors reported that consistently correlated to and predicted the rural practice. Although rural background was proved to be the single most important determinant, a study by Playford et al^15^ in Australia found that rural immersion in undergraduate medical education increased the likelihood of urban background students practising in the rural area (OR: 5.1). Compared to rural background alone, rural immersion increased urban origin graduates’ odds of rural practice by a factor of 4 (OR: 4.2). Rural immersion for two versus one year also increased the likelihood of rural background students working rurally (OR: 10.4 vs 6.6^42^; OR: 4.4 vs 2.3^50^). McGrail et al^79^ in Australia similarly found that the outcomes from rural training were increased when considered concurrently with rural origin (OR 52). A study by Rabinowitz et al^44^ in the United States found that rural origin, rural intention and practice specialty choice independently related to rural practice, and when combined, resulted in a threefold increase in the relative risk of rural practice. Playford^14^ found that without rural intention, rural immersion did not affect rural origin students’ subsequent practise location. Thus, rural background combined with rural immersion and rural intention significantly increased the odd of taking up rural practice. Differing from the finding of Rabinowitz et al, Playford’s study showed that student selection factors (rural origin and rural intention) were not enough; rural immersion was required to increase the likelihood of the selected students taking up rural practice.

**Table 3:** Summary of Factors Associated With Improved Rural Medical Workforce Recruitment, Development and Retention

**Factors Associated**	**Recruitment**	**Retention**	**Development**
Personal characteristics	Male^58,63,96^ (Japan, USA) Female^15,55,76^ (Australia)		
	Older age at school entry^43,96^ Older age graduates^14,15,63^ Early-stage career^96^	IMGs^85^	
Medical school admission criteria	Lower admission test scores^42,43^ Higher interview scores^76^ Graduate entry^40,46^ Prior tertiary experience at school entry^40^ International students^46^	Graduate entry^57^	
Rural related personal interest/career plan/career	GP^41,50^ Primary care^96^ Rural practice^14,41,44,46^ Rural generalist^76^ Family medicine^44,52,72^	Primary care^75^	
Rural background	Rural background^14,15,40-42,44,46,47,50,52,54,60,63,71,79,92,96^ Underserved background (minority/indigenous with rural background)^63,71,76,92^ Lower socio-economic level^43^	Place of birth^80^	
Participation in a targeted program	Rural immersion^15,40,47-53,55,60,70,94,96,100^ Longer term rural immersion^49,50,52,60,76^ Rural internships/vocational trainings^54,58,76,78,79,87,97^ Assured admission program^72,103^ Rural-focused medical school^61,62,64,65,67-69,73,74,104^ Rural Practitioner Program^90^ Joint GP Services^89^ Diffusion of Primary Care Team^88^ Bonded students^40,46,50,66^	Rural immersion^57^ Rural internships/vocational trainings^71,78,81,91,97^ Rural-focused medical school^67,75,95,99^ Rural Practitioner Program^90^ Joint GP Services^89^ Rural enrichment training^82^ Bonded physicians^86^	Rural internships/vocational trainings^78,91,97^ Rural Practitioner Program^90^ Joint GP Services^89^ Rural preparation workshops^83^ Rural enrichment training^77,82^

Abbreviations: GP, general practitioner; IMGs, International medical graduates.

####  University/Medical School Level

 The most dominant strategies for improved recruitment were reported at the university/medical school level, with ‘educational strategy’ the only theme derived from 36 studies.

####  Educational Strategy

 Educational strategies for improved recruitment were listed and included student selection, rural exposure/immersion, and a comprehensive medical school program approach. There are differences in the rural experience reported for the undergraduate and postgraduate medical programs. Postgraduate medical programs were undertaken entirely in a rural area (decentralised or distributed). In contrast, the rural exposure/immersion for most undergraduate programs is only a component of the duration of the entire program.^78-80,97,102^ The comprehensive medical school program was a strategy designed to increase rural workforce recruitment, comprising combinations of preferential rural student selection, rural exposure or immersion and rural curriculum with a focus on rural/remote practice.^19^

####  Student Selection

 Admission with a focus on recruitment of rural background students was described by six studies^40-45^ (excluding those using selection within a comprehensive medical school program). These studies reported waived admission test requirements, given the internationally known influence of socio-economic strata on educational attainment. They emphasised students’ commitment to serving the rural community (the United States) or fulfilling an admission quota set by the government (Australia). Data on the importance of rural background, intention to practice in rural areas, and intention to become a primary care doctor have all become key admission factors.^44,54^ One study from Australia reported an increased likelihood of rural practice for medical graduates with prior tertiary experience before starting medicine.^40^

####  Rural Exposure/Rural Immersion

 Thirteen studies reported rural exposure/immersion effects in improving rural medical workforce recruitment.^14,15,46-56^ The requirements to participate in rural clinical attachments were documented as optional and competitive (Australia).^14,15,55^ The placement settings varied from small secondary hospitals, rural general practice, or a generalist primary care doctor clinic. Australia was the only country identified where rural immersion is implemented nationwide through Rural Clinical Schools funded by the Australian government.^14,15,62,67^

####  Rural Training

 Only one study from Canada reported rural postgraduate training at the medical school level. Unlike similar programs at the government/non-government level, this postgraduate training program was established in a medical school as residency training in family medicine.^58^ The residents trained in distributed sites were 15 folds more likely to practice in rural communities, small towns, and regional centres than those trained in metropolitan teaching centres.^58^

####  Comprehensive Medical School Program

 Sixteen studies (26%) described comprehensive medical school programs.^60-76^ Those that reported a comprehensive medical school program also had a stated rural mission for their medical school. These medical schools created a specific program to implement their rural mission. They included such programmes as the Rural Physician Program in Michigan State University, USA,^69^ the Physician Shortage Area Program in Jefferson Medical College, USA,^74^ the Rockford Rural Medical Education Program, University of Illinois, USA.^72^ Some terminology used by these medical schools with the comprehensive programs were “*rurally-oriented medical school*”^42^ and “*socially-accountable, community-engaged medical school*.”^46,79“^*Rural pipeline*” was used in some studies to explain the path comprehensive medical schools provided, starting from selecting rural background students to establishing rural immersion and training for the students.^70,81^ James Cook University in Australia is one of several comprehensive medical schools that have entirely rural campuses to ensure the success of the rural pipeline model.^70^ The duration of undergraduate rural placements varied between 18 months in the Philippines^67^ and up to 24 months for Rural Clinical Schools in Australia.^60^ A compulsory rural exposure was a part of the program in the Philippines socially-accountable, community-engaged medical schools.^45,46,70^

####  Government/Non-Government Organisation Level

 At this recruitment strategy level, eight studies reported strategies for improved recruitment—two strategies were listed as an educational strategy, two financial incentives, and four multidimensional strategies.

####  Educational

####  Rural Training

 Two studies reported rural postgraduate training in the Australian context initiated by The *Australian College of Rural and Remote Medicine* (ACCRM).^78,79^ One reported that rural general practitioner (GP) vocational training associated significantly with subsequent rural practice^79^ and the other reported Rural Generalist training approach with improving service provision, recruitment and retention of staff.^78^

####  Financial Incentives

 Only 2 (3%) studies reported using a financial strategy alone to enhance rural and remote medical workforce recruitment. Although different terms were used to describe them (incentive-based scholarship and obligatory time commitment),^86,87^ there were strong similarities in the operation of the schemes, all with positive results.

####  Incentive-Based Programs, Bonded Schemes and Scholarship

 One Australian study reported bonded scholarship as a successful strategy in rural medical workforce recruitment. The New South Wales (NSW) Rural Resident Medical Officer Cadetship Program was established in 1988 by the NSW Department of Health. It aimed to increase the number of junior doctors in rural hospitals.^87^ Cadets were offered bonded scholarships and financial support for medical students during their final 2 years of undergraduate study. The return of service contract was to complete two of their first three postgraduate years in a rural NSW hospital.^87^ Cadets were subsidised to attend education and development events over their four years with the program,received a relocation allowance to assist with moving for their rural service and had access to personalised mentoring, networking and support opportunities.^87^ However, there was no documentation about a penalty if the cadets breached the scholarship agreement or withdrew from the program.^87^

####  Obligatory Time Commitment

 A study from the United States^95^ reported obligatory service schemes linked to work in underserved areas in return for concessions on international medical graduates (IMGs) J-1 visa requirements to leave the United States and return home. The waiver cancelled the return-to-home requirement on the basis that those serving in an area of workforce shortage would work in for an additional three years in an area of workforce need.^95^ This program required a 50% local match with state funds up to an annual maximum of $40 000 for up to 3 years.^95^

####  Multidimensional 

 There were four studies that reported successful multidimensional strategies toward rural medical workforce recruitment.^84,88-90^ A Chilean study reported government level involvement, offering educational, financial, management, environment and social support, and external incentives.^90^ Salary, paid training inclusive of specialisation and other training, as well as housing for rural doctors, were included in the financial strategy. Individuals’ work-related activities were scored and used in the application for the specialisation program.^90^ Facilitated recruitment (payment of tuition fees) and retention (intake into expanded essential health and education services, improved rural professional connections, and provision of internet and mobile phone connection) were all used in this multidimensional strategy.^90^ A Norway joint general practice services ^89^ successfully combine management and educational strategy to attract and retain doctors in a Norway municipal. A study from France reported improved GP density in rural areas by enforcing policy to settle Primary Care Teams within rural areas with financial support from their national government and national health insurance.^88^ A study from Brazil^84^ reported a national initiative with combined strategies that successfully improved rural doctors recruitment. The strategies included increasing supply of medical doctors in the rural by adding more places in medical courses and residency training and opening new medical schools in rural municipalities. The other strategies used were establishing fixed-term contracts to attract doctors and investment in health infrastructures.^84^

####  Multilevel (a Collaboration Between University and Government/Non-government)

 At this level, there were five studies that reported strategies listed as educational (n = 2), financial (n = 1) and multidimensional strategy (n = 2).

####  Educational

####  Student Selection

 One study from Canada^92^ reported a partnership between a provincial government and medical schools in providing places for Francophone minority students resulting in improved recruitment of doctors in rural locations in the province.^92^

####  Comprehensive Medical School Program

 A comprehensive program, namely Pukawakawa, was established as a partnership between the University of Auckland, Northland District Health Board and Hokianga Health.^94^ Key strategies of this program were student selection and rural immersion at the penultimate year of the medical course, resulting in a large proportion of graduates working in rural and regional areas.^94^

####  Financial

####  Obligatory Time Commitment

 A Japanese study documented arrangements for the publicly funded Jichi Medical University^65^which the home prefectures funded medical students for the entire six years of their undergraduate education. In return for the fee waiver, graduates are required to work in their home prefectures for nine years, including three years of postgraduate training and six years of rural service.^65^ After this nine-year obligation, Jichi Medical University graduates, can choose their workplaces freely.^65^ Graduates who breach the obligation must pay 22 600 000 yen (equivalent to 150 667 GBP) plus interest charges of 10% a year after graduation.^65^

####  Multidimensional 

 A study from the United States reported a multidimensional strategy.^98^ This strategy combined entrepreneurial, flexible discretionary grant-making and local convening capabilities of a private foundation with the comprehensive set of resources of a public university through a community-based approach successfully overcame shortages in the local healthcare delivery workforce.^98^ A study from Scotland^97^ reported a multidimensional strategy that enhances the rural medical workforce’s recruitment and retention and professional development, namely GP Rural Fellowship. This strategy combined educational (rural training), financial (shared funding), and management (service) that successfully provided an effective platform for a stimulating and supported rural professional development.^97^

###  Development

 Compared to strategies implemented to improve recruitment, strategies to enhance rural workforce development were only reported in 9 (15%) studies overall. These studies reported personal and professional strategies to develop the rural and remote workforce. Although the aim to develop rural/remote workforce was only explicitly mentioned in two of these studies, the other seven studies described practical strategies for doctors’ personal and professional development.

####  University/Medical School Level

####  Educational

####  Professional Development

 Only one study reported a strategy that improved rural medical workforce development at the university/medical school level. This strategy was listed as a professional development strategy, a specific ‘blended distance education program’ for junior doctors working in rural hospitals in India.^77^ This strategy was initiated by the medical school to equip its graduates who work in rural district hospitals. ^77^ Blended learning was implemented to assist the junior doctors with skills and clinical updates required at rural hospital level in India.^77^

####  Government/Non-Government Organisation Level

 At this level of development strategy, six studies reported strategies for improved development – three strategies listed in the educational strategy, and three in the multidimensional strategy.

####  Educational

####  Rural Training 

 This strategy was documented in a study from Australia.^78^ A “*rural training pathway*” (RTP) was the term used to explain rural training established to equip rural practitioners to return or remain their practice in rural areas. This study reported an improved professional development, besides recruitment and retention of rural workforce through sustainable implementation of RTP to Rural Generalist.^78^ This program was established by the ACCRM to deliver focussed professional development for rural and remote practitioners to maintain and enhance their skills.^78^

####  Professional Development

 Two studies from Canada^82^ and Australia^83^ reported effective professional development programs. Rural physicians’ skills enrichment program in Canada was reported to achieve development training goals and constant use of upgraded skills.^82^ In Australia, a rural vocational workshop as a part of the Rural Generalist program was reported highly valued by the participants. It provided future rural medical practitioners with professional support and networking opportunities, promoted identity formation, and stimulated rural career planning.^83^ Intentions to implement changes in practice was reported as an effect of this strategy.

####  Multidimensional

 Under this type of strategy, three studies reported effective programs in enhancing the development of the rural medical workforce. Studies of Pena (Chile)^90^ and Kehlet (Norway)^89^ have been reported under ‘recruitment strategy’, but they also reported a program providing professional development for the rural medical workforce. Straume in Norway^91^ reported decentralised internships and specialised training that combined strategies including management and education as a part of their physicians retention strategy. This strategy provided continuous medical education and counteracted professional isolation, improving health workforce retention in rural settings.^91^

####  Multilevel (a Collaboration Between University and Government/Non-government)

####  Educational 

####  Rural Immersion

 One educational strategy listed in this level was a collaboration between government and university that benefited doctors in rural hospitals with professional development. A study from the United States^93^ documented a partnership between a state government and a medical school. A vital component of this program was a longitudinal (nine-month) integrated clerkships at several rural hospitals in the state. Unlike other rural training strategies that targeted the students, this study aimed at doctors who participated as preceptors at rural hospitals. Their participation in the program was reported to increase professional and overall job satisfaction and to enhance clinical skills and medical knowledge

####  Multidimensional

 A study by MacVicar in Scotland^97^ reported a multidimensional strategy that enhances recruitment and retention and professional development of rural medical workforce, namely GP Rural Fellowship. This strategy combined educational (rural training), financial (shared funding), and management (service) that successfully provided an effective platform for a stimulating and supported rural professional development.^97^

###  Retention

 As shown in Table 2, there are limited studies that looked at the effect of rural background and rural immersion in improving retention compared to the recruitment of the medical workforce. However, participation in targeted programs reported improved retention. There were five studies purely reporting positive retention strategies to ensure *continuity in rural practice* (rural immersion and comprehensive medical school program).^57,80,95,102^ Three studies measuring retention were from Thailand.^57,95,99^ These studies evaluated a national program from different strategy perspectives (Educational– rural exposure^57^; multidimensional^99^; comprehensive medical school program^95^). Most studies documented retention for at least three years, and some reported average retention of four years^95^ or five years,^72,82,91^ with the range from 3 years to more than 14 years.^72,82,91,95^ Among studies reporting retention strategies, only one reported a low turn-over rate.^89^

####  University Medical School Level 

####  Educational

####  Rural Exposure

 Boonluksiri in Thailand^57^ reported medical schools strategy using rural exposure successfully enhanced rural medical workforce retention. More than four years of retention time was reported as an outcome of the application of longer contact time in community-based learning.

####  Rural Training

 A study from the United States^59^ reported the implementation of the Rural Training Track of Family Medicine Residency increased retention rates of the medical workforce in rural areas.

####  Comprehensive Medical School Program

 Two studies reported improved retention of the rural medical workforce as well as recruitment. Studies of MacDowell^72^ and Glasser^75^ from the United States reported a comprehensive Rural Medical Education program that encouraged their students to choose rural practice with excellent retention rates as the outcome.

####  Government/Non-government Organisation Level

####  Educational

####  Rural Training

 Two studies from Australia and one from Norway reported rural training enhanced their rural medical workforce retention. Robinson, in Australia,^80^ reported decentralised training in rural areas for GPs, resultantly in a positive influence in retaining GP in rural practice after completing the rural training. A study by Orda, from Australia^78^ reported an RTP established by the ACCRM, enhancing professional development, recruitment, and retention of the medical workforce in rural areas. Straume’s study in Norway^81^ documented over five years retention rate of family physicians and public health/community medicine physicians after rural training.

####  Professional Development

 A study from Canada^82^ reported effective professional development programs. Rural physicians’ skills enrichment program documented achievement of development training goals and constant use of upgraded skills, resulting in improved development and retention of the rural medical workforce.^82^

####  Policy

 Only one study reported policy in isolation, while other studies reported policy as a part of a multidimensional strategy. A study from Canada reported a return-of-service policy applied to IMGs who sought full licensure for practice.^85^ The policy required IMGs to work in underserviced rural areas as part of a return-of-service agreement as they seek eligibility for full provincial licensure and certification by the College of Family Physicians of Canada. Although the IMG only fulfilled the three-year return-of-service, this was seen as the best short-term solution for a long-term rural workforce shortage problem.

####  Financial Incentives

####  Obligatory Time Commitment

 As also been reported in recruitment strategy, Opoku’s^86^ study in the United States documented improved retention resulted from three years obligatory commitment in rural areas demanded from IMGs with J-1 visa in order to stay in the USA after their medical training.

####  Multidimensional

 Three studies reported a multidimensional strategy that has also been listed in recruitment and development strategy. Studies from Norway^89,91^ and Chile^90^ shared government initiatives successfully improve retention using a combination of strategies. A Norway study reported a low turn-over rate after the introduction of the program (Senjalegen Doctors).^89^

####  Multilevel (a Collaboration Between University and Government/Non-government)

####  Educational

####  Comprehensive Medical School Program

 A study from Thailand^95^ reported improved retention of the rural medical workforce as an outcome of a comprehensive medical school program supported by the national government. This study examined the program’s educational strategy apart from the program’s multidimensional nature (see below).

####  Multidimensional

 Two studies used the multidimensional strategies for improved retention of rural medical workforce. Besides a study from Scotland^97^ that has been reported in the recruitment and development section, another successful collaborative multidimensional strategy for rural workforce retention originated in Thailand.^99^

 Two government-funded initiatives were reported, the Collaborative Project to Increase Production of Rural Doctors (CPIRD) and the One District One Doctor (ODOD) program. With a primary objective of increasing doctors in rural and remote areas, these special recruitment initiatives functioned through collaboration between medical schools and Ministry of Public Health hospitals. Educational, financial, and regulatory benefits were deployed. Educational strategies included recruiting students with a rural background, utilising existing health services outside major cities as training facilities, and enabling early rural service exposure. Students recruited under the ODOD program were supported with financial incentives in addition to government institutional support. They were obligated to 12 years of rural service commitment. In contrast, the students recruited under the CPIRD scheme received no direct funding. Instead, their support was paid directly to the participating medical schools and hospitals, linked to an obligation to work for the government for three years. For the CPIRD scheme, the government enforced regulated rural placements and mandatory service with a non-adherence penalty after graduation.^99^

###  Similarities and Differences Between Approaches Implemented in HICs and LMICs

 Regarding educational strategies, medical schools in both HICs and LMICs have implemented rural student selection, rural exposure and rural-context curriculum. These medical schools also either waived or changed admission requirements for students with a rural background.

 Australia and Thailand both had detailed examples of incentive-based strategies. The Bonded Medical Places (BMP) Commonwealth initiative in Australia was similar to the CPIRD in Thailand, where a medical school place was granted for students with rural interest and practice intention pre-admission, with funding given to the participating medical schools (eg to defray the costs of rural travel for recruitment) while students also paid the tuition fee. In Australia, reports of bonded schemes included those supported by the Australian government, BMP and Medical Rural Bonded Scholarship (MRBS),^14,42,49,66,76^ Rural Australia Medical Undergraduate Scholarship^76^ and rurally oriented scholarships from regional/state government such as the Queensland Health Rural Scholarship.^76^ BMP offered a Commonwealth Supported Place (CSP) to first-year Australian medical students although the tuition fee remained the student’s responsibility, while other rurally orientated scholarship schemes provide various levels of financial funding in addition to CSP.^70^ In return for the CSP and financial assistance, all schemes required a legal contract to work in a ‘District of Workforce Shortage’ with half of the return-of-service obligation allowing graduates prevocational and vocational training to be counted.^70^ However, as these studies did not document the financial penalty for those who breach the contract, which reputedly is fairly frequent, the actual workforce impact of bonding is not known.

 ODOD program in Thailand and The Bonded Scholarships in the Australian context were similar as both are full scholarships given to students with a rural background. Return in service as an obligatory time commitment was also implemented in both income countries, with variation in the duration and the penalty obligations.

 Multidimensional approaches were also implemented in HICs and LMICs such as Chile targeted rural doctors, and in Thailand, targeted medical students. There were mixed educational, financial, and government policies to address recruitment, retention, and development of the rural medical workforce.

## Discussion

 This study aimed to synthesize international evidence of the positive impacts of programs or approaches implemented to address the problem of the medical workforce shortage. The review maps the published evidence under a series of headings related to successful strategies to improve recruitment, development, and retention of the medical workforce in rural and remote areas. Publications from both HICs and LMICs are considered concurrently to assure comprehensiveness, even though the conditions of medical training and practice differ considerably.

 No study reported using a randomised controlled trial to assess a strategy, nor was there any case-control study. This reflects the difficulties with implementing such studies in the real world, where experimental designs would result in inequitable recruitment, forced training and mandatory work for those not interested in rural practice. Therefore, this scoping review aims not to test a hypothesis but rather to explore the existing state of knowledge in an area. As Arksey and O’Malley stated, the nature of scoping review is to identify studies that have been conducted and not to assess the quality of the studies. Hence, the lack of RCT or case-control studies is not a limitation.

 For factors determining the recruitment, retention and development of the medical workforce in rural and remote areas, the most reported factors were rural background, rural exposure/immersion, and participation in a comprehensive medical school program. Although student selection factors are considered the single most important factor for rural workforce recruitment and retention, rural exposure/immersion at any stage of training also increased the likelihood of rural practice. The rural workforce ORs for these factors were consistently associated with rural practice in both low-middle income and HICs. These factors were frequently taken into account by medical schools, government and related parties when implementing programmes to improve the rural and remote medical workforce.

 We found that the strategies positively impacting recruitment, retention, and development of the rural medical workforce were educational, policy, financial incentives, and multidimensional strategies. The undergraduate educational strategy was the most commonly reported strategy with positive results. Though postgraduate training was also found to have significant results, most educational strategies internationally were implemented at the medical school-university level. Additionally, medical schools that received government financial support also had higher odds of rural workforce recruitment and retention. One positive driver for this to become more common has been the introduction and development of socially-accountable community-engaged medical schools^11,17^ as defined by the WHO.^5^ An increasing number of medical schools are now aware of their responsibilities to respond to community priority health concerns and more equitably recruit and retain a rural and remote medical workforce.

 Educational strategies with clear evidence of effectiveness are on a continuum from rural background student selection, rural exposure during medical school, and rural oriented medical school. Although rural background is predictive of rural practice, most studies using educational strategies combined them with rural immersion for the simple reason that there are many more students of urban than rural background. Given the relatively small pool of rural background students enrolled in medicine, their numbers are insufficient to comprehensively address rural medical workforce shortages. Yet even rural background students are impacted by rural immersion, doubling the odds of rural practice after rural immersion; the longer the exposure, the higher the likelihood of the graduates practising in rural and remote areas.^15,50,79^ Hence, although rural immersion programs are expensive, medical schools have benefitted from government support to implement rural placements after they have recruited an annual cohort of rural students.

 Furthermore, medical schools are also becoming more attuned to rural curriculum for all students, adding this to student selection and rural exposure as their strategy to improve rural workforce recruitment. These medical schools work under a socially accountable/rural pipeline rubric and consider themselves to be community engaged. These include Northern Ontario School of Medicine – Canada,^68^ University of Manila-School of Health Sciences - Philippines,^62^ Jefferson Medical College – USA,^74^ and James Cook University – Australia.^70^

 As a result of comprehensive strategies that include rural recruitment, rural exposure, and rural curriculum, there is now a formal network of medical schools from different countries and incomes to raise awareness of medical schools’ social accountability for rural and remote communities.^105^ This network enables medical schools from low-income countries to implement a similar strategy to high-income counterparts with great success.^105^ These partnerships occur between medical schools and research institutes in underserved and rural regions of HICs and LMICs (Australia, Canada, US, UK, Netherlands, Ghana, Malawi, South Africa, the Philippines, Nepal, Sudan).^106^

 More coercive strategies reported such as bonded scholarships and obligatory time commitment in rural areas have been met with some success. Examples of this strategy come from Australia and Thailand. In Australia, the Bonded Medical Program was established in 2001 and included two schemes: BMP (offered to those who otherwise would not meet the requirements for admission) and rural-based MRBS. Students supported by these schemes were more likely to join regional, rural, and remote practices (OR range 1.63-4.21).^14,49,66,76^ However, the MRBS program has been discontinued, and its quarantined places given instead to the BMP programme, under the new name: Bonded Return of Service System effective from 2020. This form of bonding continues to provide students with a CSP in a medical course at an Australian university in return for a commitment to work in eligible regional, rural and remote areas for a specified period after completion of their medical course. As a deterrent from defecting from the scheme, postgraduate repayment of the Commonwealth contribution to the university during the medical training will be required if there is a breach of return of service agreement. Thailand has two bonded schemes similar to those implemented in Australia, one with a scholarship and the other a bonded place scheme. As already described, the CPIRD offers no financial support for the students besides its contribution to the medical schools, and the ODOD program that provides scholarships including tuition fees and living allowances.

 Recruiting IMGs has been one government level strategy that proved to be effective in rural medical workforce recruitment. Example from Canada,^85^ the United States,^86^ and Australia demonstrated that IMG doctors are more likely to work and be retained in rural and remote areas and therefore government policies have been issued to recruit IMG’s specifically as rural and remote workforce. However, long term dependency on doctors trained in relatively low-income countries needs to be re-assessed. In Australia there is now substantial funding allocated through schemes directed at undergraduate and postgraduate pathways to develop sufficient locally trained doctors for rural Australia.

 Chile and Thailand’s multidimensional strategies provide good examples of what can be achieved with a holistic approach. Although there are some differences in terms of the target of the programs – Chile for the doctors, and Thailand for medical students, both countries are similar in using a wide range of strategies. Educational, incentive-based and regulation enforcement approaches were implemented, and have improved the recruitment and retention of the rural and remote medical workforce in both countries. Besides the enforcement, these countries also improved the working environment to better suit the needs of rural doctors to develop and thrive.

 There is overwhelming evidence that both HICs and LMICs can implement similar strategies and programs despite their differing local contexts and challenges. Although more evidence and ongoing evaluation is required from LMICs, strategies which work internationally include a positive bias for rural origin students, rural exposure during medical school, and commitment to rural curriculum work in all contexts. These should be considered by both HICs and LMICs aiming to improve recruitment and retention of their rural and remote medical workforce. These strategies are also in line with the WHO global policy recommendation to improve retention of rural and remote health workers.^5^ This scoping review provides evidence that there are many effective strategies feasible for worldwide implementation, despite the wide differences in socio-economic factors that are often given as a reason for reluctance to change. However, while adjustment and adaptation to match approaches to the local context are required, considerable will and action are needed at all levels of governance and government to improve the rural medical workforce shortage.

 We identified some important gaps through this scoping review. There are relatively limited studies discussing retention (n = 16) and development (n = 9) of rural and remote medical workforce compared to studies that focus on recruitment strategies (n = 50). Furthermore, discussion about rural and remote medical workforce within these studies is limited with respect to the ongoing approaches that will lead doctors to remain as a committed rural medical workforce. Australia appears to lead the way through the formation of ACCRM, established in 1997, specifically dedicated to rural doctors as college members, with unique needs, interests and skills.^107^ The development in 2019 of national “rural generalist training” speaks to the same strength in Australia. This sustained work shows that collegiality, focussed professional development, and quarantined funding for rural practitioners can powerfully maintain and enhance rural and remote doctors’ skills, competence and ability to thrive in rural practices.^107^

 As to comparisons, as a good number of studies did not define their use of the “rural” or “rural background,” it is difficult to come to definitive international conclusions. Even studies published in the same country have used different standards of classification. For example, in Australia, there are three standardised classifications. The Australian Standard Geographical Classification – Remoteness Areas, the Modified Monash Model, and Rural, Remote and Metropolitan Area classification. Likewise, with the term rural background, there are differences by country, and the term has evolved over time. This situation makes it challenging to undertake comparative studies across countries, within a single country, and even for longitudinal studies within one institution over time. The United Nation recommendation method for delineating rurality^37^ can be used to promote international comparison. Further studies are needed to assist the project in evaluating whether similar strategies have similar effects within a country, regional, and globally, irrespective of geographical descriptors. This is particularly important for doctors in remote practice who comprise a unique cadre of “rural” doctors.

 Our results were based on the success of the program or strategy implementation regardless of the definition of rurality used in a study, and discretion is needed in adapting the successful strategies used in other countries. However, within the concept of recruitment, development, and retention, the evidence was supported internationally, where the strategies were successful across different contexts. Therefore, they are likely potential to be adapted in other countries.

 Although this scoping review included searched literature from a range of databases with the expectation of comprehensively capturing relevant health literature, there is a possibility that the database did not contain all the available literature, potentially limiting our findings for a global perspective.

 As we deliberately omitted studies reporting on negative outcomes of interventions, some valuable insights presented in studies of “failed” recruitment/development/retention initiatives will be missed from our review. A future investigation could be considered to augment the findings from this review.

## Conclusion

 This scoping review concluded that rural background and rural exposure with participation in a rural-focused medical school were the main determinant factors of recruitment and retention of the rural and remote medical workforce. Educational strategies, ie, student selection, rural learning experience, and integrative rural-focus curriculum in medical schools, successfully improve rural and remote medical workforce recruitment and retention. We have shown that this evidence is strong across international contexts, with significant probabilities and a higher likelihood of rural practice. There are similarities and differences between approaches implemented in HICs and LMICs. However, the strategies we have reported as successfully implemented in the countries studied have the potential to be more widely implemented with positive outcomes; further studies to investigate their practicality in other countries and contexts will provide further evidence.

## Acknowledgments

 The authors would like to acknowledge Ms. Terena Solomons, Medical Librarian (University of Western Australia) who has provided invaluable expertise to the development and refinement of the search strategy of this scoping review.

## Ethical issues

 This scoping study is a part of a project with several studies that have been granted Ethical Approval from The University of Western Australia No. RA/4/20/5065.

## Competing interests

 Authors declare that they have no competing interests.

## Authors’ contributions

 FN led the design and conceptualisation of this work, drafted the protocol, developed the search strategy, and conducted the search, data extraction, analyse, discuss and conclude the study. SC, KF, ST, DP, and RC were involved in the conceptualisation of the review design, specifically in establishing the review question as well as the inclusion and exclusion criteria, provided feedback on the manuscript and copy-edited the manuscript. SC, ST, RC, and DP guided the conceptualisation and design of the study and data analyses and have revised all drafts of this manuscript for important intellectual content and clarity. All authors approve the publishing of this manuscript.

## Supplementary files


Supplementary file 1. PRISMA Checklist.

Supplementary file 2. Search Strategy.

Supplementary file 3. Charting Table.

Supplementary file 4. Included Studies With Contexts, Strategies and Outcomes.

Supplementary file 5. Definition of Rurality Per Country.
