# Supplementary material for: Strategies to Facilitate Improved Recruitment, Development, and Retention of the Rural and Remote Medical Workforce: A Scoping Review
Source: Int J Health Policy Manag. 2021 Nov 15;11(10):2022–37. doi: 10.34172/ijhpm.2021.160 (PMC9808272; doi:10.34172/ijhpm.2021.160)
Supplement: Supplementary file 2 — Search Strategy. [file ijhpm-11-2022-s002.pdf]

**Article title:** Strategies to Facilitate Improved Recruitment, Development, and Retention of the Rural and Remote Medical Workforce: A Scoping Review

**Journal name:** International Journal of Health Policy and Management (IJHPM)

**Authors' information:** Farah Noya[1]*, Sandra Carr[1], Kirsty Freeman[2,1], Sandra Thompson[3], Rhonda Clifford[4], Denese Playford[5]

[1]Division of Health Professions Education, School of Allied Health, University of Western Australia, Perth, WA, Australia.
[2]Duke National University Singapore Medical School, Singapore, Singapore.
[3]Western Australian Centre for Rural Health, The University of Western Australia, Perth, WA, Australia.
[4]School of Allied Health, University of Western Australia, Perth, WA, Australia.
[5]The Rural Clinical School of WA, School of Medicine, The University of Western Australia, Perth, WA, Australia.

(*Corresponding author: farah.noya@research.uwa.edu.au)

**Supplementary file 2.** Search Strategy

Database: Ovid MEDLINE(R) and Epub Ahead of Print, In-Process & Other Non-Indexed Citations, Daily and Versions(R) <1946 to March 15, 2019>

Search Strategy:

1. *Rural Population/
2. *Rural Health Services/
3. *Rural Health/
4. rural area*.ti,ab.
5. rural communit*.ti,ab.
6. rural location*.ti,ab.
7. rural practice*.ti,ab.
8. remote area*.ti,ab.
9. remote communit*.ti,ab.
10. remote location*.ti,ab.
11. remote practice*.ti,ab.

12. underserved area*.ti,ab.

13. underserved location*.ti,ab.

14. underserved communit*.ti,ab.

15. geographically isolated area*.ti,ab.

16. geographically isolated communit*.ti,ab.

17. island* communit*.ti,ab.

18. small island* communit*.ti,ab.

19. remote island* communit*.ti,ab.

20. poorly served area*.ti,ab.

21. poorly served communit*.ti,ab.

22. underserviced area*.ti,ab.

23. "rural and remote area*".ti,ab.

24. *Medically Underserved Area/

25. 1 or 2 or 3 or 4 or 5 or 6 or 7 or 8 or 9 or 10 or 11 or 12 or 13 or 14 or 15 or 16 or 17 or 18 or 19 or 20 or 21 or 22 or 23 or 24

26. *general practitioners/ or *physicians, family/ or *physicians, primary care/

27. *general practice/ or *family practice/

28. exp Medical Staff/

29. medical doctor.mp.

30. Medical officer*.ti,ab.

31. medical worker*.ti,ab.

32. medical profession*.ti,ab.

33. medical workforce.ti,ab.

34. medical graduate*.ti,ab.

35. health centre*.ti,ab.

36. medical centre*.ti,ab.

37. international medical graduate*.ti,ab.

38. foreign medical graduate*.ti,ab.

39. communit* medicine.ti,ab.

40. *Workforce/

41. *Physicians/sn, sd

42. 26 or 27 or 28 or 29 or 30 or 31 or 32 or 33 or 34 or 35 or 36 or 37 or 38 or 39 or 40 or 41

43. *personnel selection/ or *"personnel staffing and scheduling"/ or *personnel turnover/ or *staff development/ or *strikes, employee/ or *work engagement/ or *workplace/

44. *Job Satisfaction/

45. *Personnel Loyalty/

46. *Personal Satisfaction/

47. *Career Choice/

48. *Career Mobility/

49. personnel recruitment.ti,ab.

50. sustainable rural practice.ti,ab.

51. (sustain* adj3 employ*).ti,ab.

52. (attract* adj3 employ*).ti,ab.

53. personnel shortage*.ti,ab.

54. workforce shortage*.ti,ab.

55. "attract and retain".ti,ab.

56. "recruit and retain".ti,ab.

57. "recruitment and retention".ti,ab.

58. "recruiting and retaining".ti,ab.

59. (workforce adj3 maldistribut*).ti,ab.

60. under distribut*.ti,ab.

61. (commit* adj3 employ*).ti,ab.

62. *Motivation/ph, sn [Physiology, Statistics & Numerical Data]

63. (intrinsic adj3 motivat*).ti,ab.

64. (hire* adj3 staff).ti,ab.

65. improv* access.ti,ab.

66. engag* employ*.ti,ab.

67. interest* employ*.ti,ab.

68. (attract* adj3 employ*).ti,ab.

69. encourage* employ*.ti,ab.

70. work* satisfaction*.ti,ab.

71. (career adj3 advance*).ti,ab.

72. Unmet Need*.ti,ab.

73. workforce need*.ti,ab.

74. recruitment strateg*.ti,ab.

75. (retention adj2 strateg*).ti,ab.

76. career development.ti,ab.

77. (plan* adj5 workforce).ti,ab.

78. 43 or 44 or 45 or 46 or 47 or 48 or 49 or 50 or 51 or 52 or 53 or 54 or 55 or 56 or 57 or 58 or 59 or 60 or 61 or 62 or 63 or 64 or 65 or 66 or 67 or 68 or 69 or 70 or 71 or 72 or 73 or 74 or 75 or 76 or 77

79. *health plan implementation/ or *health priorities/

80. *Health Policy/

81. government initiative*.ti,ab.

82. support structure.ti,ab.

83. practical model*.ti,ab.

84. (rural adj3 package*).ti,ab.

85. alternative model*.ti,ab.

86. locum service*.ti,ab.

87. locum support.ti,ab.

88. (compulsory adj3 assignment*).ti,ab.

89. compulsory service*.ti,ab.

90. bond* scheme*.ti,ab.

91. bond* service*.ti,ab.

92. vacancy rate*.ti,ab.

93. utilization of service*.ti,ab.

94. duration of service*.ti,ab.

95. *Program Evaluation/

96. *Survival Analysis/

97. *regression analysis/

98. factor* impact*.ti,ab.

99. polic* analysis.ti,ab.

100. polic* initiativ*.ti,ab.

101. *physician incentive plans/ or *"salaries and fringe benefits"/

102. *Remuneration/

103. financial incentive*.ti,ab.

104. financial inducement*.ti,ab.

105. monetary incentive*.ti,ab.

106. (non-financial adj3 inducement*).ti,ab.

107. non-monetary incentive*.ti,ab.

108. incentiv* measure*.ti,ab.

109. incentiv* polic*.ti,ab.

110. *Socioeconomic factors/

111. exp Education, Medical/

112. faculty development.ti,ab.

113. professional development.ti,ab.

114. rural exposure.ti,ab.

115. rural learning experiences.ti,ab.

116. rural scholarship.ti,ab.

117. *Training Support/

118. educational grant*.ti,ab.

119. *Schools, Medical/

120. community participation.ti,ab.

121. social accountabilit*.ti,ab.

122. *Social Responsibility/

123. *Community-Institutional Relations/
