# Supplementary material for: Strategies to Facilitate Improved Recruitment, Development, and Retention of the Rural and Remote Medical Workforce: A Scoping Review
Source: Int J Health Policy Manag. 2021 Nov 15;11(10):2022–37. doi: 10.34172/ijhpm.2021.160 (PMC9808272; doi:10.34172/ijhpm.2021.160)
Supplement: Supplementary file 3 — Charting Table. [file ijhpm-11-2022-s003.pdf]

Article title: Strategies to Facilitate Improved Recruitment, Development, and Retention of the Rural and Remote Medical Workforce: A Scoping Review

Journal name: International Journal of Health Policy and Management (IJHPM)

Authors' information: Farah Noya[1]*, Sandra Carr[1], Kirsty Freeman[2,1], Sandra Thompson[3], Rhonda Clifford[4], Denese Playford[5]

[1]Division of Health Professions Education, School of Allied Health, University of Western Australia, Perth, WA, Australia.
[2]Duke National University Singapore Medical School, Singapore, Singapore.
[3]Western Australian Centre for Rural Health, The University of Western Australia, Perth, WA, Australia.
[4]School of Allied Health, University of Western Australia, Perth, WA, Australia.
[5]The Rural Clinical School of WA, School of Medicine, The University of Western Australia, Perth, WA, Australia.

(*Corresponding author: farah.noya@research.uwa.edu.au)

Supplementary file 3. Charting Table

	**Description**
**Article details**	
Title	
Authors	
Abstract	
Published Year	
Journal Volume Issue Pages	
Accession Number	
DOI	
Covidence #	
Country	
**Study details**	
Article type	
Design Methods	
Analysis	
Group	
Participants	
Response rate	
Instrument/tools	
Inclusion criteria	

Exclusion criteria	
Group differences	
Intervention (Comparator/control group)	
Power/significance of the study findings	
Predictors	
Target outcomes	
Study outcomes	
**Study context**	
Recruitment/development/retention	
Level of approach	
Type of strategy/initiative (educ, finance, etc)	
Substrategy	
**Details of the program**	
Name	
Participants	
Program delivery	
Facilitators	
Duration of the program	
Evaluation mechanism	
Timing of evaluation	
**Definition of rurality**	
