# Supplementary material for: Strategies to Facilitate Improved Recruitment, Development, and Retention of the Rural and Remote Medical Workforce: A Scoping Review
Source: Int J Health Policy Manag. 2021 Nov 15;11(10):2022–37. doi: 10.34172/ijhpm.2021.160 (PMC9808272; doi:10.34172/ijhpm.2021.160)
Supplement: Supplementary file 4 — Included Studies With Contexts, Strategies and Outcomes. [file ijhpm-11-2022-s004.pdf]

Article title: Strategies to Facilitate Improved Recruitment, Development, and Retention of the Rural and Remote Medical Workforce: A Scoping Review

Journal name: International Journal of Health Policy and Management (IJHPM)

Authors' information: Farah Noya[1]*, Sandra Carr[1], Kirsty Freeman[2,1], Sandra Thompson[3], Rhonda Clifford[4], Denese Playford[5]

[1]Division of Health Professions Education, School of Allied Health, University of Western Australia, Perth, WA, Australia.

[2]Duke National University Singapore Medical School, Singapore, Singapore.

[3]Western Australian Centre for Rural Health, The University of Western Australia, Perth, WA, Australia.

[4]School of Allied Health, University of Western Australia, Perth, WA, Australia.

[5]The Rural Clinical School of WA, School of Medicine, The University of Western Australia, Perth, WA, Australia.

(*Corresponding author: farah.noya@research.uwa.edu.au)

Supplementary file 4. Definition of Rurality Per Country

	**Definition of rurality**
**High income countries**	
Australia	Australian Standard Geographical Classification – Remoteness Areas (ASGC–RA). The classification consists of five categories: RA1 – Major Cities, RA2 – Inner Regional, RA3 – Outer Regional, RA4 – Remote and RA5 – Very Remote. Rural = ASGC-RA 2-5[14, 15, 40-43, 47, 48, 50, 54, 55, 70, 76, 79, 87, 100]

	Definition of rurality
	Australia’s Modified Monash Model (MMM) classification, which defines rural as MMM 2–7 (MMM-2: > 50 000 population; MMM-3: 15–50 000; MMM-4: 5–15 000; MMM-5: < 5 000; MM6–7: remote and very remote [46, 49, 60, 83]
	Uses two classification [47, 78]<br>1. ASCG-RA 2-5<br>2. Rural, Remote and Metropolitan Area classification (RRMA) 4 and above. ***RRMA is the previous classification used in Australia 2009 and beyond, replaced by ASGC-RA.***
Canada	Urban (10 000 or greater population) or rural (less than 10 000 population) based on **2001 census population**, based on Statistics Canada metropolitan influence scores. [101]
	Eight Communities with populations ranging from 1600 to 16 000 people, located 70–600 km from the major cities of Sudbury or Thunder Bay, and serving catchment areas with populations up to 35 000. (defined by the informants)[65]
	Statistics Canada Postal Code Conversion Files. Practices located in census metropolitan areas (CMAs) and census agglomerations (Cas) were considered urban. CMAs and Cas have populations of at least 100 000 and 10 000, respectively. All areas outside of CMAs and Cas were classified as rural.[68]
	By size of community and by a description of the primary population served: inner city, urban, regional center (small city with high access to specialists) and rural/small town (population under 10 000 with primarily family physician care). These categories were chosen to be consistent with those used in a periodic national physician survey[58]
	General Practice Rurality Index-Simplified (GPRI-S)[92]
	Any Alberta community more than 50 km outside a major metropolitan centre.[82]

	**Definition of rurality**
	Communities with a population of 25 000 or less were classified as rural, communities with a population between 25 000 and 200 000 were classified as regional and communities with a population greater than 200 000 were classified as urban.[53]
Chile	No documentation[90]
France	Multidimensional indicators (Primary health care delivery; Population: demographics, health status, household, and socioeconomic status; Spatial structure). 18 indicators with data comes from multiple sources: census data (National Institute of Statistics and Economic Studies, INSEE), morbidity-mortality data (French National Institute of Health and Medical Research, CépiDc-INSERM), accessibility of GP (Institute for Research and Information in Health Economics, IRDES), and ambulatory HHRs supply data from the NHI (Caisse Nationale d'Assurance maladie, CNAMTS). This resulted in 6 types of rural areas: (**1.** Growing suburban areas with lower accessibility of GPs and nurses, **2.** Privileged areas regarding health and socioeconomic status with average level of accessibility of GPs, **3.** Industrial and agricultural areas with lower accessibility of primary care, **4.** Deprived areas in terms of social and health status with lower accessibility of primary care, **5.** Tourist and attractive areas with socioeconomically deprived populations and better accessibility of primary care, **6.** Remote areas with older population)[88]
Japan	The rurality of the communities was determined by population density quintiles. "Quintile 1" (the group with the lowest population densities) to "quintile 5" (the group with the highest densities). Each quintile contained 20% of the all the municipalities. The cut-off values for the quintiles were 48.5, 125.2, 297.8, and 840.5 persons per square kilometre
New Zealand	Based on Regional Rural Admission Scheme at the University of Auckland. Any DHBs largely outside of Auckland, Hamilton, Tauranga, Wellington, Porirua, Hutt, Upper Hutt, Christchurch or Dunedin City Councils are considered rural or regional.[94]
Norway	No specific definition[89, 91, 102]

	Definition of rurality
	The municipalities are divided into four centrality levels (levels 0 – 3) by population, central functions and distance to an urban settlement. This means that the municipalities closest to the urban settlement are placed on the same level as the urban settlement. Municipalities on level 3 are regional centres in contrast to municipalities on level 0 that are the most remote. Rural communities in this study are understood as municipalities on level 0 or 1.
Scotland	The Scottish Government's urban–rural classification includes definitions of both 'accessible' and 'remote rural: *Remote Rural*: Less than 3000 people and with a drive time of over 30 minutes to a settlement of 10 000 or more. *Remote Small Towns*: Between 3000 and 10 000 people and with a drive time of over 30 minutes to a settlement of 10 000 or more. *Accessible Rural*: Less than 3000 people and within 30 minutes' drive of a settlement of 10 000 or more.[97]
USA	US Department of Agriculture Economic Research Service classification: small rural 2500 population and intermediate rural 2500–10 000 population)[51]
	Rural Urban Continuum Codes (RUCC) and nonmetropolitan RUCC codes as a surrogate to identify rural/small town practice. The RUCC categorizes counties into 9 classifications, 3 as metro and 6 as non-metro. The 6 non-metro classifications were used as a proxy for rural practice. In Kentucky, 3 of the non-metro counties had urban populations of approximately 28,000, and the majority had towns of only 4,000-9,000.[52]
	Rural–Urban Density Typology (RUDT). The RUDT classification system uses the population density thresholds of the U.S. Census Bureau's classification system, the U.S. Office of Management and Budget's urban population nucleus requirements, and other criteria to classify counties as "rural," “mixed rural,” “mixed urban,” and “urban.” The RUDT classification identifies rural and urban counties based on (1) the percent of the population that is rural or urban, (2) the population in urbanized areas, and (3) population density.[44, 73, 74]

	Definition of rurality
	**Definition of rurality**
	The Rural–Urban Commuting Area code 2.0 (RUCA), which are designed to define the level of rurality based on census information. The RUCA codes range from 1 to 10 with subcategories. A zip code considered rural if the RUCA code was 4 or higher and nonrural if the RUCA code was less than 4. [63, 69, 72]
	Subcategories: rural county with a population of less than 60 000, and rural county with a population of more than 60 000 people.[75]
	Not documented[59]
**Low-Middle income countries**	
Brazil	Not documented[84]
India	India's secondary hospitals, which are small hospitals mainly located in rural areas. These hospitals have between 20 and 200 beds, with a generalist practice of medicine, surgery, paediatrics, and obstetrics and gynaecology; limited laboratory support; and local community based programs such as antenatal care and tuberculosis programs.[77]
The Philippines	Not documented clearly. Implied: The Eastern Visayas region of The Philippines [67]<br>Study objective terms: Population size <100,000 [62]
Thailand	Not documented. Implied: Rural areas in Thailand[57], Rural provinces in Thailand[99] Rural districts are classified by geographical distance from a city or town, low population density and low revenue generation. There are 878 rural districts with 742 community hospitals throughout Thailand.[95]
