# Supplementary material for: Strategies to Facilitate Improved Recruitment, Development, and Retention of the Rural and Remote Medical Workforce: A Scoping Review
Source: Int J Health Policy Manag. 2021 Nov 15;11(10):2022–37. doi: 10.34172/ijhpm.2021.160 (PMC9808272; doi:10.34172/ijhpm.2021.160)
Supplement: Supplementary file 5 — Definition of Rurality Per Country. [file ijhpm-11-2022-s005.pdf]

Article title: Strategies to Facilitate Improved Recruitment, Development, and Retention of the Rural and Remote Medical Workforce: A Scoping Review

Journal name: International Journal of Health Policy and Management (IJHPM)

Authors' information: Farah Noya[1]*, Sandra Carr[1], Kirsty Freeman[2,1], Sandra Thompson[3], Rhonda Clifford[4], Denese Playford[5]

[1]Division of Health Professions Education, School of Allied Health, University of Western Australia, Perth, WA, Australia.

[2]Duke National University Singapore Medical School, Singapore, Singapore.

[3]Western Australian Centre for Rural Health, The University of Western Australia, Perth, WA, Australia.

[4]School of Allied Health, University of Western Australia, Perth, WA, Australia.

[5]The Rural Clinical School of WA, School of Medicine, The University of Western Australia, Perth, WA, Australia.

(*Corresponding author: farah.noya@research.uwa.edu.au)

Supplementary file 5. Included Studies With Contexts, Strategies and Outcomes

Context (Recruitment/ development/retention)	Type of strategy/initiative	Substrategy	Program Name	Study	Country	Study outcomes
Recruitment	Educational	Integrative/comprehensive (more than 1 strategies)	RCSWA	Playford 2017	Australia	In multivariate analysis, only being of older age at graduation (OR 2.28 (95% CI 1.40, 3.72), P = 0.001), being from a rural background (OR 2.99 (95% CI 1.85, 4.85), P < 0.001), being a recipient of a Medical Rural Bonded Scholarship (OR 3.36 (95% CI 1.68, 6.73, P = 0.001) and participating in the RCSWA remained significantly related to rural work (OR 3.10 (95% CI 1.95, 4.93), P < 0.001).
			Rural oriented/focused medical School	Woolley 2016	Australia	Attending the JCU clinical school in the main town was a strong predictor of JCU MBBS graduates currently practising (2014) in the Townsville, Cairns and Mackay health service districts (p<0.001, POR=2.9; p<0.001, POR=3.8; p=0.033, POR=3.6; respectively).

Context (Recruitment/ development/retention)	Type of strategy/initiative	Substrategy	Program Name	Study	Country	Study outcomes
			Duration of immersion and selection criteria	McGrail 2018	Australia	Longer duration (18-24 vs 12 months) of rural training (relative risk ratio, RRR, 3.37, 1.89-5.98) and completing both schooling and training in the same rural region (RRR: 4.47, 2.14-9.36) were associated with returning to practice in the same rural region after training
			Rural oriented/focused medical School	Woolley 2014	Australia	Multivariate logistic regression identified that the likelihood of JCU MBBS graduates practising in a rural location in PGY 5 was predicted by rural background (having a hometown at application categorised as an ‘outer regional’ or ‘remote’ location) (P<0.001; POR =3.9), having Aboriginal or Torres Strait Islander heritage (P= 0.031; POR=5.6)
			Memorial’s pathways to rural practice (rural generalist focused)	Rourke 2018	Canada	The percentage of all Memorial PG graduates (19.0%) and FM PG graduates (26.9%) practicing rurally was significantly better than the national average for PG (6.4%, p<0.000) and FM (12.9%, p<0.000).
			Socially accountable medical school	Mian 2017	Canada	Positive changes, linked to collaboration with NOSM, included achieving a full complement of physicians in 5 communities with previous chronic shortages of 30%-50% of the physician supply, substantial reduction in recruitment expenditures, decreased reliance on locums and a shift from crisis management to long-term planning in recruitment activities. The magnitude of positive changes varied across communities, with individual leadership and communities' active engagement being key factors in successful physician recruitment

Context (Recruitment/ development/retention)	Type of strategy/initiative	Substrategy	Program Name	Study	Country	Study outcomes
			NOSM rural pipeline in medical education	Wenghofer 2017	Canada	Physicians who graduated from NOSM-UG were more likely to have practices located in rural Ontario (OR = 2.57; p = 0.014) whereas NOSM-PG physicians were more likely to have practices in northern Ontario (OR = 57.88; p < 0.001).
			PUKAWAKAWA A regional rural program	Matthews 2015	New Zealand	In 2013, 62% were working in rural or regional areas, with 31% in the Northland DHB.
			Socially accountable, community engaged medical school	Halili 2017	Phillipines	ADZU-SOM medical graduates were more likely to work in their first position as a Rural/Municipal Health Officer than were comparator school graduates (p ¼ 0.001). ADZUSOM medical graduates were also more likely to be working in a Government tertiary hospital in their current position as a generalist Medical Officer/Resident/Consultant (p < 0.001) or working in a Rural Health Unit (p < 0.001) as Municipal Health Officers (p ¼ 0.003), while comparator school graduates were more likely to currently working in private hospitals (p ¼ 0.033) or Government specialist hospitals (p ¼ 0.040), often as surgical (p ¼ 0.010) or nonsurgical/medical specialists (p < 0.001).

**Context (Recruitment/ development/retention)**	**Type of strategy/initiative**	**Substrategy**	**Program Name**	**Study**	**Country**	**Study outcomes**
			Socially accountable, community engaged medical school	Woolley 2018	Phillipines	Thirty-one percent of ADZU-SOM medical graduates practised in communities <100 000 population versus 7% of graduates from the conventional school in the Zamboanga region (p<0.001), while 61% of SHS-Palo medical graduates practised in communities <100 000 population versus 12% of graduates from the conventional school in the Visayas region (p<0.001). Twenty-seven percent of ADZU-SOM graduates practised in lower income category communities (categories 2-6) versus 8% of graduates from the conventional school in the same region (p<0.001), while 49% of SHS-Palo graduates practised in lower income category communities (categories 2-6) versus 11% of graduates from the conventional school in the same region (p<0.001).
			Socially-accountable, community-engaged medical education	Siega-Sur 2017	Phillipines	SHS-Palo graduates were also more likely to work in rural and remote areas (p < 0.001). Graduates also stayed longer in both their first medical position (p = 0.028, 3.7 years) and their current position (p < 0.001, 6.8 years), although not clearly in rural/remote posts
			Physician Shortage Area Program (PSAP)	Rabinowitz 2011	USA	PSAP graduates were much more likely both to practice rural family medicine than their non-PSAP peers (32.0% [31/97] versus 3.2% [65/2,004]; relative risk [RR] 9.9, confidence interval [CI] 6.8 –14.4, P.001) and to practice any specialty in rural Pennsylvania (PSAP 24.7% [24/97] versus non-PSAP 2.0% [40/2,004]; RR 12.4, CI 7.8 –19.7, P .001).
			Rural track clerkship (RTC)	Quinn 2011	USA	Over 57% of students who participated in the RTC program (and potentially other MU-RTPP offerings) chose a rural location for their first practice.

Context (Recruitment/ development/retention)	Type of strategy/initiative	Substrategy	Program Name	Study	Country	Study outcomes
			MS with a rural mission: Rural family Medicine	Fuglestad 2017	USA	Age| >31 years Vs <31 years OR 2.56 (CI 1.61–4.05) p<.001. Hometown |Rural/Urban OR 1.59 (CI 1.26–2.01) p <.001. Hometown*Age (Interaction) OR 3.65 (1.38–9.63) p=.009. Men were 1.33 times more likely than women to select rural practices, but this difference was narrowing (Figure 2), perhaps related to increasing numbers of women from rural hometowns. Being older and having a rural hometown increased the odds of rural practice, and these two effects were synergistic, such that older rural hometown graduates were 3.65 times more likely to choose a rural practice than younger urban hometown graduates (Table 3). Older AI graduates were particularly likely to go into rural practice, and overall 47% of AI graduates chose rural practice.
			Rural Physician Program (RPP)	Wendling 2016	USA	RPP graduates were more likely to practice a rural high-need specialty (RPP 122/168 [73%]; MSU-CHM 1,612/2,610 [62%]; P = .006), and practice in a rural area (RPP 76/168 [45%]; MSU-CHM 361/2,610 [14%]; P < .001).
		rural exposure	Rural exposure	Runge 2016	Australia	Rural practice was associated with a rural childhood (odds ratio (OR) (95% confidence interval, CI) 1.89 (1.10, 3.27) P = 0.02) and any time spent as an intern (OR 4.07 (2.12, 7.82) P < 0.001) or registrar (OR 4.00 (2.21, 7.26) P < 0.001) in a rural location. Physicians with a rural childhood and rural training were most likely to be in rural practice. However, those who had a metropolitan childhood and a rural internship were approximately five times more likely to be working in rural practice than physicians with no rural exposure (OR 5.33 (1.61, 17.60) P < 0.01). The findings demonstrate the positive effect of rural vocational training on rural practice.

Context (Recruitment/ development/retention)	Type of strategy/initiative	Substrategy	Program Name	Study	Country	Study outcomes
			Rural immersion + rural practice setting	O'Sullivan 2018	Australia	The adjusted odds of working in a rural area were significantly increased if students were immersed for one full year (odds ratio [OR], 1.79; 95% confidence interval [CI], 1.15-2.79), for between 1 and 2 years (OR, 2.26; 95% CI, 1.54-3.32) and for 2 or more years (OR, 4.43; 95% CI, 3.03-6.47) relative to no rural immersion. The strongest association was for immersion in a mix of both regional hospitals and rural general practice (OR, 3.26; 95% CI, 2.31-4.61), followed by immersion in regional hospitals only (OR, 1.94; 95% CI, 1.39-2.70) and rural general practice only (OR, 1.91; 95% CI, 1.06-3.45). More than 1 year's immersion in a mix of regional hospitals and rural general practices was associated with working in smaller regional or rural towns (<50 000 population) (relative risk ratios [RRR] 2.97; 95% CI, 1.82-4.83)
			Rural Clinical School WA	Playford 2014	Australia	Using logistic regression, RCSWA participation had a strong relationship with working rurally (rural-background RCSWA graduates: odds ratio [OR], 7.5; 95% CI, 3.5-15.8; urban-background RCSWA graduates: OR, 5.1; 95% CI, 2.9-9.1). Rural background without RCSWA participation (OR, 4.2; 95% CI, 1.8-9.2) and older age (age in 2012, 30-39 years: OR, 2.2; 95% CI, 1.3-3.7 v >/= 40 years: OR, 6.6; 95% CI, 2.8-15.0) were also significant factors for working rurally.

Context (Recruitment/ development/retention)	Type of strategy/initiative	Substrategy	Program Name	Study	Country	Study outcomes
			Longitudinal rural clerkship (1 academic year)	Playford 2015	Australia	The two groups not participating in the RCSWA had 45.5% and 52.4% of subjects in outer regional/very remote locations, respectively. In comparison, 78.7% of those who had participated in the RCSWA were currently practicing in outer regional/very remote locations. When the 3 groups were compared, the significant predictors of working in a more remote practice compared to working in an inner regional area were being female (OR 1.75 95% CI 1.13, 2.72, P = 0.013) and participating in the RCSWA (OR 4.42, 95% CI 2.26, 8.67, P < 0.001). In multivariate logistic regression that corrected for gender and remoteness of rural address before entry to medical school, participation in the RCSWA still predicted a more than 4-fold increase in the odds of practicing in a more remote area (OR 4.11, 95% CI 2.04, 8.30, P < 0.001).
			RCSWA	Playford 2017	Australia	Rural background (OR, 3.91; 95% CI, 2.12-7.21; P < 0.001) and experience in an RCS (OR, 1.93; 95% CI, 1.05-3.54; P = 0.034) were significant predictors of rural practice in the multivariate analysis of all potential factors. When interactions between intention, origin, and RCS experience were included, RCS participation significantly increased the likelihood of graduates with an initial rural intention practising in a rural location (OR, 3.57; 95% CI, 1.25-10.2; P = 0.017).
			Socially accountable, community engaged medical school	Woolley 2017	Australia	Forty-seven (9%) of JCU Bachelor of Medicine and Bachelor of Surgery graduates in the first seven cohorts had practised for at least 1 year in a remote location between PGY 4 and 10. Practice in a 'remote' town was predicted by undertaking rural generalist training (p<0.001; prevalence odds ratio (POR)=17.0), being awarded an 'above average' interview score at medical school selection (p=0.006; POR=5.1), attending the Darwin clinical school

Context (Recruitment/ development/retention)	Type of strategy/initiative	Substrategy	Program Name	Study	Country	Study outcomes
						in years 5–6 (p=0.005; POR=4.7), being female (p=0.016, POR=3.6) and undertaking an outer-regional or remotely based internship (p=0.006; POR=3.5). CART analysis identified Indigenous graduates as another key subgroup of remote practice graduates.
			Rural clinical school	Shires 2015	Australia	Students who had spent a year at the UTAS RCS were five times more likely to be working in RA3 to RA5 than those who hadn't spent a clinical year there (28% vs 7%, $\chi2$ (1) =59.5, p<0.0001) (odds ratio (OR) 4.9, 95% confidence interval (CI) 3.2– 7.6). Using the Modified Monash Model, it was found that UTAS RCS graduates were nine times more likely (OR 9.0, 95%CI 4.7– 17.2) to be working in the regional cities and smaller towns of Tasmania.
				Kwan 2017	Australia	Independent predictors of LTRP (odds ratio [95% confidence interval]) were RB (2.10 [1.37-3.20]), RCS-1 (2.85 [1.77-4.58]), RCS-2 (5.38 [3.15-9.20]), GP (3.40 [2.13-5.43]), and bonded scholarship (2.11 [1.19-3.76]). Compared to being single, having a metropolitan background partner was a negative predictor (0.34 [0.21-0.57]). The effects of RB and RCS were additive - compared to MB and MCS (Reference group): RB and RCS-1 (6.58[3.32-13.04]), RB and RCS-2 (10.36[4.89-21.93]).
			Rural Clinical School	Moore 2018	Australia	The percentage of graduates working in rural areas was highest in the later postgraduate years – PGY6 and above. More than twice as many rural stream graduates were working in RA2–5 locations in PGY6–11 (34.7%) than in PGY1–5 (16.1%; = 10.73, P <0.001).

Context (Recruitment/ development/retention)	Type of strategy/initiative	Substrategy	Program Name	Study	Country	Study outcomes
			Longitudinal Integrated Clerkship (LIC)	Campbell 2019	Australia	After accounting for key covariates, LIC participants who had additional rural training of >6 weeks in years 3 and/or 5 of the course (group A) had the strongest odds of working in rural areas (OR 5.04, 95%CI 2.80–9.09). In contrast, LIC participants for whom LIC was their only rural training (group B) were no more likely to take up rural practice than the metropolitan-only group (OR 1.66, 95%CI 0.75–3.68). Among the non-LIC participants with year 4 rural training, students with longer rural exposure (>1 year in years 3 and/or 5 of the course, group C) also had higher odds of working in rural locations than students with shorter rural training (=1 year, group D) (OR 3.68, 95%CI 2.58–5.23 and OR 2.39, 95%CI 1.48–3.87, respectively) Students who had participated in the LIC group with additional rural training in years 3 and/or 5 of the course (group A) had the strongest odds of working in smaller regional or rural towns (population size<50 000) (OR 5.62, 95%CI 2.81–11.20). Students in this group (group A) also had strong odds of working in large regional centres (=50 000 population) as did the non-LIC year 4 rural group (group C) (OR 4.11, 95%CI 1.32–4.95 and OR 4.49, 95%CI 2.81–7.19, respectively). Overall, rural work was consistently positively associated with rural background, being an international student and having a BMP or MRBS return of service obligation, but negatively associated with being in a later career stage (=PGY 7) (Table 4). Working in a smaller rural town was positively associated with being a graduate entry student or having an interest in rural practice when commencing medical school.

Context (Recruitment/ development/retention)	Type of strategy/initiative	Substrategy	Program Name	Study	Country	Study outcomes
			Rural Clinical School	McGirr 2019	Australia	Students with a rural background were 4.1 times more likely to be practising in a rural location according to ASGC ($p<0.001$) and students who participated in extended RCS placement were 1.9 times as likely to be practising in a rural location ($p<0.001$). After rural background was controlled for, students who attended an RCS were 1.6 times more likely to be in rural practice ($p=0.004$). After extended RCS placement was controlled for, students with a rural background were 3.8 times more likely to be practising in a rural location ($p<0.001$). According to the MMM (3–7), students with a rural background were 3.1 times more likely to be in rural practice than students with a metropolitan background ($p<0.001$). Students who participated in extended RCS placement were 3.1 times as likely to be practising in a rural location ($p<0.001$). After rural background was controlled for, students who participated in extended RCS placement were 2.6 times as likely to be practising in a rural location ($p<0.001$). After extended RCS placement was controlled for, students with a rural background were 2.6 times more likely to be practising in a rural location ($p<0.001$).
			Longitudinal Integrated Clerkship (LIC)	Myhre 2016	Canada	A 3x2 χ^2 test examining type of clerkship and practice locations was significant (Pearson $\chi^2 2 = 11.85$, $p = 0.003$). Subsequently, we performed three 2x2 pairwise comparisons of practice location and clerkship stream. The significance level was adjusted to 0.017 ($\alpha= 0.05/3$). The association between type of clerkship and rural versus urban practice location was significant (Pearson $\chi^2 1= 9.56$, $p = 0.002$)

Context (Recruitment/ development/retention)	Type of strategy/initiative	Substrategy	Program Name	Study	Country	Study outcomes
			Free clinic participation	Petrany 2017	USA	Participants were more likely to practice in rural areas (63%, 27 of 43) than residents who did not participate (43% [41 of 95], P = .033).
				Crump 2016	USA	Practicing in a rural location occurred in only 7% of standard campus graduates, but the proportion increased to 45% of rural campus graduates. The adjusted OR indicates that choosing a rural practice location was significantly higher for graduates participating in the rural campus (OR = 5.46) compared with standard program graduates. Rural practice location was significantly associated with having a rural upbringing (OR = 2.67), as well as choosing a family medicine residency (OR = 5.08)
			Longitudinal Integrated Clerkships in Rural Communities	Bing-You 2014	USA	Doctors (as preceptor) job satisfaction increased (overall, professional, academic component of job) and their own ongoing education (clinical skills, base of medical knowledge) was improved by having inquisitive learners working with them.
		Rural training (Postgraduate)		McGrail 2016	Australia	Generalised estimating equation logit models identified a highly significant association between rural training pathways and subsequent rural practice that was sustained for 5 years after vocational registration; it was substantially strengthened when combined with rural origin (cohort 2 v cohort 4: odds ratio [OR], 24; 95% CI, 13e43; cohort 1 v cohort 4: OR, 52; 95% CI, 24e111).

Context (Recruitment/ development/retention)	Type of strategy/initiative	Substrategy	Program Name	Study	Country	Study outcomes
			Distributed family medicine training	Jamieson 2014	Canada	In bivariate analysis, site of training was correlated with practice location/population served, with those training in the Metro Vancouver based sites more likely to serve an urban or inner-city population and those in the distributed sites more likely to serve a regional or rural population (Fig2). When regional and rural practice populations are combined, the relationship between training site and practice location is even more pronounced (Fig3). This relationship persists at 5 and 10 years after graduation (χ 2=31 and 33 respectively, df=1, p
		Student selection		Ray 2015	Australia	Graduates having either a rural or a remote home town at application were more likely to practise in rural (RA 3–5) towns than graduates from metropolitan/inner regional centre across all postgraduate years. For example, the prevalence odds ratios (POR) for graduates practising in a rural town at postgraduate year 1 (PGY 1) having either a rural or remote hometown were 2.6 and 1.8, respectively, times that of graduates having a metropolitan/inner regional hometown, while at PGY 9 the PORs had increased to 4.2 and 9.5, respectively. Bonded medical place students showed lower engagement in rural practice in the 5 years of data available.
			rural background students	Puddey 2015	Australia	In logistic regression, those practicing in a rural location in 2014 were more likely to have come from the lower 6 IRSAD deciles (OR 2.75, 95% CI 1.44, 5.23, P = 0.002), to be older (OR 1.86, 95% CI 1.09, 3.18, p = 0.023) and to have a lower UMAT-3 (Non-verbal communication) score (OR 0.98, 95% CI 0.97, 0.99, P = 0.005). After further controlling for either rural background or RCS participation, only age and UMAT-3 remained as independent predictors of current rural practice

Context (Recruitment/ development/retention)	Type of strategy/initiative	Substrategy	Program Name	Study	Country	Study outcomes
				Hogenbirk 2015	Australia	Logistic regression found that rural versus urban background was a significant predictor of rural (outside major city) first practice location (odds ratio (OR) 5.0, 95% confidence interval (CI) 1.3-19.2) and rural current practice location (OR 5.6, 95% CI 1.5-21.2) for fully qualified doctors. General practitioner versus other medical specialists significantly predicted first (OR 7.2, 95% CI 2.1-25.2) or current (OR 3.6, 95% CI 1.1-11.9) rural practice location. Preference for a rural practice location in 5-10 years was predicted by rural background (OR 4.4, 95% CI 1.6-11.8) and positive intention towards rural practice upon completing MBBS (OR 4.6, 95% CI 1.7-12.6). Surveyed in 2011, 28% of those who also responded to the 2006 survey shifted their preferred future practice location from rural to urban communities versus 13% shifting from urban to rural (McNemar-Bowker test, P = 0.02).
			Graduate Entry and Non-standard Entry	Playford 2019	Australia	In the univariate analysis, both NSE students and GE students had an increase in the odds of practicing rurally compared to SLE students (OR 3.89, 95% CI 2.29-6.60, p<0.001 and OR 2.22, 95% CI 1.42-3.46, p<0.001, respectively). Within the GE students, those from health/allied health or humanities backgrounds had an increase in the odds of practicing rurally compared to SLE students, while those from biological science/science or physical sciences did not. The associations with rural background and RCS participation were additive and of a similar magnitude to those we have reported in previous cross-sectional surveys [6.24]. There was a significant association with being a recipient of a bonded medical rural scholarship (OR 3.98, 95% CI 2.07-7.65, p<0.001) but not a bonded medical place. Students in the lower 8 IRSAD deciles were more likely to be practicing rurally (OR 2.71,

Context (Recruitment/ development/retention)	Type of strategy/initiative	Substrategy	Program Name	Study	Country	Study outcomes
						95% CI 1.8-4.04, p<0.001) and those born overseas were less (OR 0.55, 95% CI 0.34-0.89, 0.001). After block entry of rural background and rural clinical school participation, being born overseas, socioeconomic status, and being in receipt of a bonded rural scholarship into a multivariate logistic regression model, subsequent entry of student group indicated that both NSE students and GE students still had increased odds of being in rural practice relative to SLE students.
			Francophone minority students	Beauchamp 2013	Canada	A rural background (of the Francophone doctors) was positively associated with the establishment of a first medical practice in a rural community. This relationship was only significant among family physicians. There was no statistically significant relationship between rurality of community of origin and rurality of current community of practice among either family or specialty physicians.
				Rabinowitz 2012	USA	A logistic regression showed that all three predictors were independently related to rural practice (P .001). Of graduates with all three predictors, 45% (45/99; CI 35%–55%) practiced in rural areas; of those with two predictors, 33% (48/145; CI 25%–41%) practiced rural; of those with one predictor, 21% (42/198; CI 15%–27%) were rural; and of graduates without any predictors, only 12% (37/320; CI 8%–15%) practiced in rural areas (Figure 1). Compared with the reference group of graduates with no predictors, the RR of practicing rural was 3.9 (CI2.7–5.7, P .001) for those with three predictors, RR 2.9 (CI 2.0 –4.2, P .001) for those with two predictors, and RR 1.8 (CI 1.2–2.8, P .01) for those with one predictor.

**Context (Recruitment/ development/retention)**	**Type of strategy/initiative**	**Substrategy**	**Program Name**	**Study**	**Country**	**Study outcomes**
	Financial incentives	Obligatory time commitment	Full scholarship at Jichi Medical University	Matsumoto 2010	Japan	Multivariate analysis showed that service experience in the communities of the first and second highest quintiles of rurality was associated with choosing such places after contract, independent of known predictors of rural practice, such as having a rural background and primary care specialty choice
		Bonded scheme scholarship	The New South Wales Rural Resident Medical Officer Cadetship Program	Lewis 2016	Australia	Over half of the cadets (n=74, 53%) were working in rural areas (RA2–5) in 2014 (Table 4) and practice location was significantly (p <0.001) influenced by career choice. The cadets with rural backgrounds were more likely to choose general practice than those from urban backgrounds. A similar analysis of cadets comparing geographic background and practice location showed cadets of rural background were more likely to be working in a rural location than cadets of urban background
	Multidimensional	Educational, financial, management		Reid 2019	USA	• Recruiting a health workforce has been successful. In the past 3 years, the County's two community hospitals have recruited from UNM programs five family physicians, two physician assistants, two dental hygienists and one emergency medical technician. • Expanding specialist consult capacity by using University telemedicine technologies. The local hospital physicians now access a range of specialist consultations, from neonatologists to neurosurgeons. • Reducing the County's exceedingly high teen pregnancy rate. UNM provided research guidance in developing a new school-based clinic and trained local providers in insertion of Long-Acting Reversible Contraceptives. • Reversal of a preconception among University students that a rotation in Lea County was undesirable. Lea County clinical rotations are now considered highly preferable. • Encouraging collegiality and collaboration within the local medical community. The

Context (Recruitment/ development/retention)	Type of strategy/initiative	Substrategy	Program Name	Study	Country	Study outcomes
						convening capability of the Foundation, combined with the politically neutral standing of the University, provided a platform for collaboration. A highly fractious medical community came together, through the facilitation of University leaders, and collaborated in new and unexpected ways. • Disseminating the model created in Hobbs to other rural communities in New Mexico. The University–Foundation partnership resulted in a new understanding of how to extend valuable University resources to rural communities to address community-driven priorities.
	Policy	Group practice in Primary Care	Primary Care Team (PCT)	Chevillard 2019	France	A slowdown in decrease of GP Density in rural areas. PCT's impact is only significant in disadvantaged deprived areas. In such areas (clusters 3 and 4), there was a negative and comparable trend over the period 2004–2008. This trend persists during the following period 2008–2012, but with a significant slowdown, at the 5% level, in treated areas. Overall, treated areas gained an average of 3.5 GPs per 100,000 inhabitants between the two periods compared with those of control areas.
		Provision of MD in rural, remote and socially vulnerable areas	Mais Medicos	Pereira 2016	Brazil	75% reduction of municipalities with <0.1 doctors/1000 population
Development	Professional Development (Educational)	Maintenance training/professional development	The Rural Generalist Vocational Preparation Workshop)	Martin 2019	Australia	One hundred per cent of participants said they would recommend this workshop to others and 88.1% reported intention to implement changes to practice. Changes were primarily around pursuit of career options, engagement with team members, development of leadership skills and improved networking. The overall workshop was rated on a 5-point Likert scale (very poor, poor, average, good, very good), 66.7% (n = 34) of the participants rated it as very good, with the remaining participants (33.3%, n = 17)

Context (Recruitment/ development/retention)	Type of strategy/initiative	Substrategy	Program Name	Study	Country	Study outcomes
						rating it as good. All participants agreed workshop facilitators were approachable, gave clear explanations, provided useful feedback and used effective teaching strategies.
			The Fellowship in Secondary Hospital Medicine (FSHM)	Vyas 2014	India	This evaluation study provides evidence that the distance developed for mobile phone use and being pilot tested by learning modules, supported by contact sessions, helped FSHM students to enable them to access information in time junior doctors develop the knowledge and skills to practice effectively in rural hospitals in India, based on the perspectives of the students and the faculty. The junior doctors felt that the blended program helped them to provide improved patient care and they gave specific examples of cases they could manage after going through the program. Based on suggestions made, the distance learning modules have been reviewed and updated; new modules have been included. In addition, the FSHM program has been created on CMC's learning management system, through which the students can access resource materials. This is currently being developed for mobile phone use and being pilot tested by FSHM students to enable them to access information in time and on the go.
Retention	Educational	rural exposure	Decentralised GP Training	Robinson 2013	Australia	Quantitative: There was a significant relationship between the place of birth and remaining in rural practice, with almost three-quarters (73% n=16) of the Australian-born respondents and almost one-quarter (23% n=8) of the overseas-born respondents remaining in rural practice after fellowship ($\chi^2 = 13.68$ p<0.001); Qualitative: for Australian and overseas-born doctors, both groups agree on what sustains them in rural practice once that decision is made: • a preference for a rural lifestyle and a fondness for

Context (Recruitment/ development/retention)	Type of strategy/initiative	Substrategy	Program Name	Study	Country	Study outcomes
						rural people and communities • the diversity and challenge of rural practice • the ability to provide holistic patient care.
			Community-based Learning	Boonluksiri 2018	Thailand	CPIRD doctors work in rural areas at a higher rate than normal track physicians (62.3% and 49.0%, P < 0.001). CPIRD retained at a higher rate (1514 of 2098 doctors; 72.1%) than normal track (4260 of 7919 doctors; 53.8%; P < 0.001). In doctors wirh Rural background: CBL, geographic location of workplace, and graduate entry were significantly associated with retention.
		Rural training (Postgraduate)	compulsory group tutorage in decentralised general practice/family medicine (FM) and public	Straume 2010	Norway	In total, 65-67% of the physicians from the programs are still working in the county 5 years after completion of the group tutorial. Rural practice provides good learning conditions when accompanied by appropriate tutelage, and in-service training allows the trainees and their families to 'grow roots' in the remote area while in training. The group tutorial develops peer support and professional networks to alleviate professional isolation. (PH: some doctors have stayed for 14 years or more, while others have stayed for shorter periods. For 15 of the doctors, more than 5 years had passed since the completion of the three mandatory years of tutelage. Of these, 10 are still working in Finnmark, comprising a five-year retention rate of 67%. During the observation period discussed, the vacancy rates for primary care physicians in Finnmark has improved.

Context (Recruitment/ development/retention)	Type of strategy/initiative	Substrategy	Program Name	Study	Country	Study outcomes
			Rural training track residency programs	Morken 2018	USA	Of the 26 physicians invited to participate in our survey, we received responses from 19 (73.1%). Nearly three quarters (73.7%) of respondents were practicing in rural areas. Of the physicians who had ever practiced in rural areas, 87.5% were still practicing in rural areas. Half of the respondents were employed at their original practice sites. None of the physicians started practicing in an urban area and moved to a rural area. Most physician mobility occurred between rural practices, and the 4 respondents who planned on leaving their current practice in the next 3 years intended to continue practicing in rural areas.
		Integrative/comprehensive (more than 1 strategies)	Collaborative Project to Increase Production of Rural Doctor	Pagaiya 2015	Thailand	The retention rate at rural hospitals was 29% for the CPIRD doctors compared to 18% for those from the normal track. Survival curves indicated a dramatic drop rate after 3 years in service for both groups, but normal track individuals decreased at a faster rate. Multivariate Cox proportional hazards modelling revealed that the normal track doctors had a significantly higher risk of leaving rural areas at about 1.3 times the CPIRD doctors. The predicted median survival time in rural hospitals was 4.2 years for the CPIRD group and 3.4 years for the normal track. The normal track doctors had a significantly higher risk of leaving public service at about 1.5 times the CPIRD doctors.

Context (Recruitment/ development/retention)	Type of strategy/initiative	Substrategy	Program Name	Study	Country	Study outcomes
	Multidimensional	Educational, financial, management/regulati on	1. Collaborative Project to Increase Production of Rural Doctor (CPIRD), 2. One District One Doctor (ODOD)	Arora 2017	Thailand	The overall and year-by-year retention of medical graduates under the special recruitment track was higher than the normal track (overall retention of 78.2% and 52.5% respectively, p<0.05). Compared to their normal track counterparts, medical graduates under the special recruitment scheme were about 2.4-fold more likely to remain working in the MOPH health services for a minimum period of 3 years (odds ratio 2.44, 95% confidence interval 2.19-2.72). Among 4869 medical graduates under the special recruitment track who remained working for the MOPH, 4425 (90.9%) still worked in the provinces to which they were primarily assigned. factors that were independently associated with 3-year retention in MOPH health services included training track, sex and geographic region
	Policy	Recruitment of IMG		Mowat 2017	Canada	Most IMGs (63.5%) remained in Manitoba, and 59.2% of this group practised outside of Winnipeg. Of those remaining in Manitoba, most (69.6%) held full provincial licensure and national certification. The regression model was significant (chi²₄ = 13.94, p = 0.007), explaining 10% of the variance in retention. Two predictors were significant: years since program graduation and Manitoba residency at the time of application.
Combination						
Recruitment, Retention	Financial incentives	Obligatory time commitment	Visa J1 waiver and State loan repayment	Opoku 2015	USA	Quant: Factors associated with retention: State loan repayment, age, primary care provider, per capita income, County unemployment rate. Comparative: Longer length of stay--> state loan repayment (among all physician and among physicians that work more than 3 years.

Context (Recruitment/ development/retention)	Type of strategy/initiative	Substrategy	Program Name	Study	Country	Study outcomes
	Educational	Integrative/comprehensive (more than 1 strategies)	Rural Medical Education (RMED) program	MacDowell 2013	USA	RMED graduates were 14.4 times more likely than non-RMED graduates to choose family medicine; 6.7 times more likely to choose a primary care practice specialty; 17.2 times more likely to be currently practicing in a rural location; and 12.8 times more likely to be currently practicing in a primary care shortage zip code. Analysis of current RMED graduates' practice locations indicates that 41.9% were within 90 miles of their fourth-year preceptorship community. Among RMED graduates practicing in Illinois, 62.1% and 73.3% were located within 60 and 90 miles, respectively, of their hometown. In terms of length of time in practice, the mean number of years is 5.3 years, with a range from 0.5 to 11.3 years. A total of 110 (68.8%) have remained in their original practice location. For these, the mean length of time in the community is 4.3 years, with a range of 0.58 to 10.6 years.
			Rural Medical Education (RMED) Program	Glasser 2010	USA	Most rural physicians in this study decided to practise in rural areas because of family ties. Eighty per cent of the physicians participating in the interviews mentioned no negative personal or family factors related to their community of practice. Outcome data on graduates from the rural medical education programme are encouraging. Over 70% opt for primary care and rural practice. Over 80% have remained in their original rural practice location. 75 (70%) are in practice in rural areas. The percentage of graduates going into rural primary care slightly increases from 64 to 70% when comparing longer term to short-term programme graduates.
Recruitment, Retention, Development	Educational	rural training (Postgraduate)	Rural generalist training	Orda 2017	Australia	Since 2013 RMO locum rates have been <1%. Registrars on the ACRRM pathway and Interns increased from 0 to 7 positions each in 2015, with similar achievements in SMO staffing.

**Context (Recruitment/ development/retention)**	**Type of strategy/initiative**	**Substrategy**	**Program Name**	**Study**	**Country**	**Study outcomes**
	Multidimensional	Educational, financial, management	the Rural Practitioner Programme.	Pena 2010	Chille	Recruitment: exceed the number of available positions by at least 2.5 times and have increased in the past three years; since 2002, acceptance rates are 100% Retention: Open positions are normally filled in subsequent calls at national or decentralized level, resulting in very few positions left vacant for more than one year. The programme, however, is less successful in motivating doctors to stay for the maximum period (6 years), achieving only 58% of the maximum length of stay. Incentive to worksite: The Rural Practitioner Programme has successfully motivated physicians to engage in non-clinical work, particularly in promoting continuous medical education and health education activities. Despite representing a large share of the total score (28 points, 40%), the least successful areas were participating in community outreach activities, developing community and health-care projects and assuming management functions. Satisfaction: more than 90% considered their experience as positive and 69.7% planned to practise as a specialist in the referral hospital. Respondents identified relationships with family and partner, working conditions, income and social relations as the most influential factors in their daily experience as rural doctors.
		Management and education	Joint GP Services: The Senja Doctor	Kehlet 2015	Norway	Significant reduction in turnover rate. Senjalegen has contributed to the improvement and continuity of GP services to the inhabitants of Tranøy (municipality).
		Educational, financial, management	Rural Fellowship	MacVicar 2016	Scotland	A total of 46 graduates of the Fellowship in the period surveyed (71%) were working in rural areas or accessible small towns in Scotland, 39 in substantive general practice roles (60%).

Context (Recruitment/ development/retention)	Type of strategy/initiative	Substrategy	Program Name	Study	Country	Study outcomes
Retention, Development	Multidimensional	Management and education	Decentralised internship and specialist training	Straume 2010	Norway	Relevant Changes: Almost twice as many medical interns as expected now take their first fully licensed job in the north of Norway. The post-training retention of primary care physicians after 5 years currently stands at 65%.
	Professional Development (Educational)	Maintenance training/professional development	Rural physicians' skills enrichment program	Gorsche 2012	Canada	All 33 enrichment participants completed their goal attainment survey, and 32 (97%) achieved their training goals. That is, they reported the achievement of training goals to be at or greater than expected, and importantly all were using their new or upgraded skills. After 5 years, all 29/29 (100%) physicians in the matched enrichment group remained in rural practice compared with 22/29 (71%) physicians who did not partake in the EP: RR=1.31; confidence interval=1.06–1.62; $P < 0.05$. Five of the seven controls, including one specialist, lost from rural practice were in practice less than 5 years (Table 2). Six of seven in the control group who left were practising in towns of 10 000 or less.
